# KSHV but not MHV-68 LANA induces a strong bend upon binding to terminal repeat viral DNA

**DOI:** 10.1093/nar/gkv987

**Published:** 2015-09-30

**Authors:** Rajesh Ponnusamy, Maxim V. Petoukhov, Bruno Correia, Tania F. Custodio, Franceline Juillard, Min Tan, Marta Pires de Miranda, Maria A. Carrondo, J. Pedro Simas, Kenneth M. Kaye, Dmitri I. Svergun, Colin E. McVey

**Affiliations:** 1 Instituto de Tecnologia Química e Biológica António Xavier, Universidade Nova de Lisboa, Oeiras 2700-157, Portugal; 2 European Molecular Biology Laboratory, Hamburg Unit, EMBL c/o DESY, Hamburg 22607, Germany; 3 Departments of Medicine, Brigham and Women's Hospital and Harvard Medical School, Boston, MA 02115, USA; 4 Instituto de Microbiologia e Instituto de Medicina Molecular, Faculdade de Medicina, Universidade de Lisboa, Lisboa 1649-028, Portugal

## Abstract

Latency-associated nuclear antigen (LANA) is central to episomal tethering, replication and transcriptional regulation of γ2-herpesviruses. LANA binds cooperatively to the terminal repeat (TR) region of the viral episome via adjacent LANA binding sites (LBS), but the molecular mechanism by which LANA assembles on the TR remains elusive. We show that KSHV LANA and MHV-68 LANA proteins bind LBS DNA using strikingly different modes. Solution structure of LANA complexes revealed that while kLANA tetramer is intrinsically bent both in the free and bound state to LBS1–2 DNA, mLANA oligomers instead adopt a rigid linear conformation. In addition, we report a novel non-ring kLANA structure that displays more flexibility at its assembly interface than previously demonstrated. We identified a hydrophobic pivot point located at the dimer–dimer assembly interface, which gives rotational freedom for kLANA to adopt variable conformations to accommodate both LBS1–2 and LBS2–1–3 DNA. Alterations in the arrangement of LBS within TR or at the tetramer assembly interface have a drastic effect on the ability of kLANA binding. We also show kLANA and mLANA DNA binding functions can be reciprocated. Although KSHV and MHV-68 are closely related, the findings provide new insights into how the structure, oligomerization, and DNA binding of LANA have evolved differently to assemble on the TR DNA.

## INTRODUCTION

*Herpesviridae* are a large family of dsDNA viruses infecting many mammalian species and are a leading cause of human viral diseases. There are eight known herpesviruses that infect humans, among which are two γ-herpesvirus family members, the Epstein-Barr virus (EBV; HHV-4) and the Kaposi's sarcoma-associated herpesvirus (KSHV; HHV8). KSHV is a γ2-herpesvirus, classified together with murine γ-herpesvirus 68 (MHV-68; MuHV-4), herpesvirus samiri (HVS) and rhesus rhadinovirus (RRV) (1). KSHV diverged from γ1-herpesviruses, such as Epstein-Barr Virus (EBV), some 100 000 years ago (2). KSHV is not pathogenic in healthy individuals but is highly oncogenic in HIV-1-infected and immunosuppressed individuals. It is involved in the pathogenesis of Kaposi's sarcoma (KS), primary effusion lymphoma and multicentric Castleman's disease. (3–5). MHV-68 is genetically similar to other γ-herpesviruses and readily infects mice, thus providing a mouse model for studying viral pathogenesis (6), while both EBV and KSHV fail to infect small laboratory animals. Like other herpesviruses, KSHV and MHV-68, have two distinct forms of infection: latency and lytic replication. During latency the virus genome persists as a nuclear multicopy circular plasmid (episome). The latent phase of infection is linked with γ-herpesvirus associated pathologies and drives the proliferation and survival of tumor cells (7).

**Figure 1.**
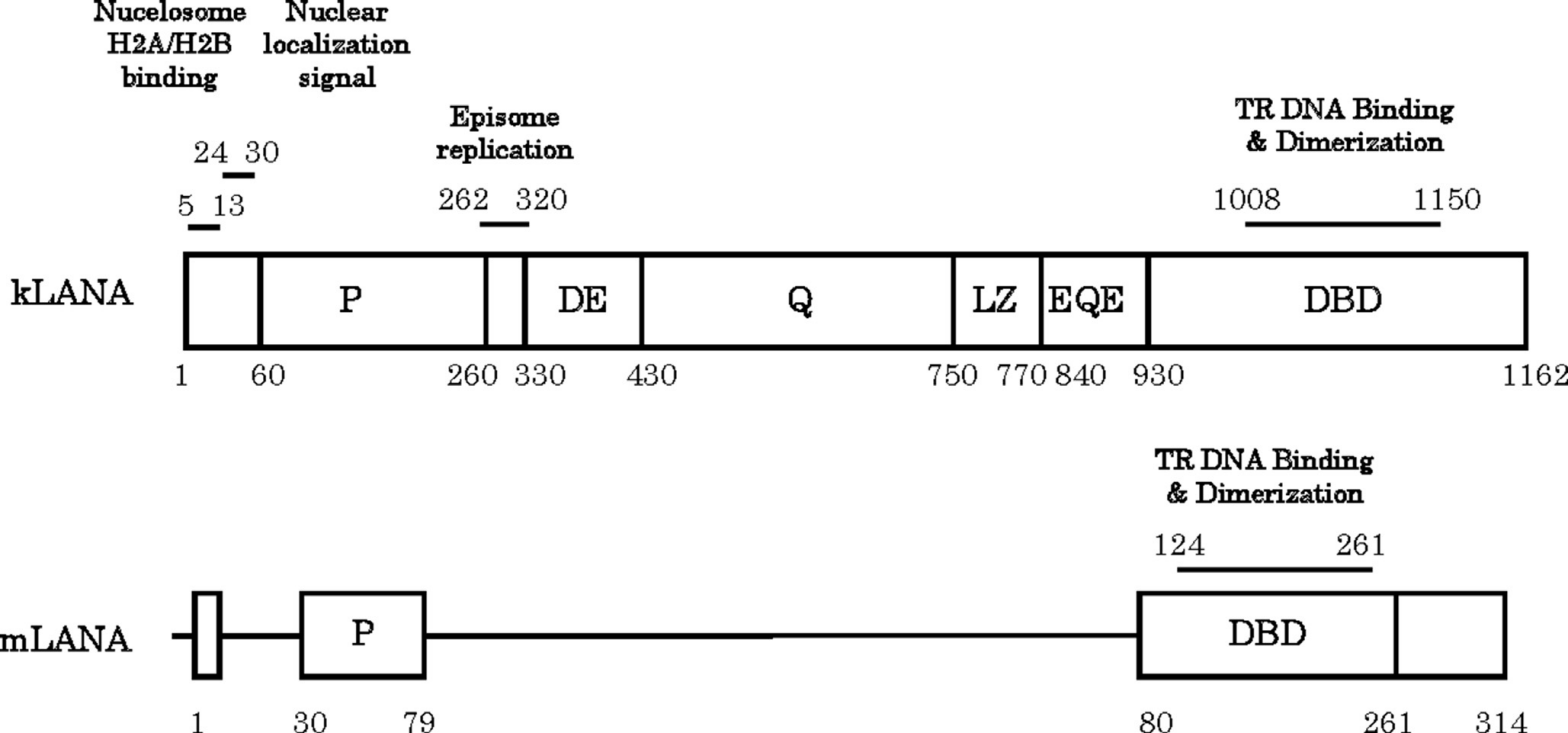
Schematic diagram of KSHV LANA (kLANA) and MHV-68 LANA (mLANA). The proline rich region (P) and the C-terminal region harboring the DNA binding domain (DBD) shared by both proteins are indicated. The DNA binding domain also mediates dimerization and interdimer association of LANA proteins. Amino acids of kLANA 5–13 mediate chromosome association through binding to histones H2A/H2B. The internal region of kLANA is absent from mLANA and comprises aspartate-glutamate (DE), glutamine (Q), and glutamine-glutamate (EQE) regions, and a putative leucine zipper (LZ) region. All these contain repeat elements. The internal unique region of kLANA, comprising amino acids 262–320, is required for episomal replication.

Among the very few genes expressed during latency, the latency associated nuclear antigen 1 (kLANA) is the dominant protein expressed in all KSHV latently infected cells (8). LANA is required for the replication and maintenance of the viral episomes and thus is essential for latency. kLANA directs the replication and tethers the viral episome to mitotic chromosomes to ensure efficient segregation of the newly replicated molecules during mitosis. kLANA consists of 1162 amino acid residues divided into at least four unique regions (Figure 1), comprising a proline-rich region, a central acidic repeat region, a putative leucine zipper, and a dimerization and viral DNA binding domain (DBD) (9). The N-terminal region is flexible and intrinsically disordered, likely linked to its promiscuous binding, while the C-terminal region is structurally ordered and stable. The first 32 N-terminal amino acid residues interact with histones H2A/H2B on the nucleosome surface (10–13). The distal C-terminus DBD is self-sufficient for both dimerization and DNA-binding. The DBD binds to specific sequences present within the terminal repeat (TR) of the viral episomal DNA, termed as LANA binding site (LBS). In KSHV, so far three LBS (kLBS-1, -2 and -3) have been identified with varying binding affinities to LANA protein. Each LBS region is able to bind one LANA dimer, containing two recognition half-sites. The LBS sequences are present in tandem within TR DNA and thus require LANA to oligomerize and interact with LBS in a cooperative manner (14–18). The central region of kLANA contains several repeat sequences which vary in length among KSHV isolates (19). In contrast, the homolog MHV-68 LANA (mLANA) protein consists only of 314 amino acid residues, lacking the acidic repeat and glutamine-rich sequence; yet mLANA is also able to efficiently tether the viral DNA and maintain viral episomes (20). mLANA binding sites (mLBS) have also been identified in MHV-68 TR DNA (21).

In addition to LANA's tethering function, both kLANA and mLANA are required to initiate episome replication by recruiting various cellular replication proteins and are also involved in activation and repression of transcription (22–24). LANA requires intrinsic flexibility in its structure and employs strategies such as oligomerization and post-translational modification to do its multiple functions. To efficiently maintain the viral episomes, two minimal replicator elements (MRE) containing the LBS sequence are required to be separated by 801 bps, irrespective of the DNA (25,26). This indicates that a spatial arrangement is critical for accommodating a super molecular structure that incorporates LANA bound to TR DNA, in addition to other host cell proteins. Such an assembly could give rise to the characteristic LANA speckles observed in mitotic KSHV infected cells.

The C-terminal DBD of LANA from KSHV and MHV-68 share a similar structural topology revealed by the crystal structures reported by us and others (27–29). The LANA DBD dimerizes by forming a central eight-stranded anti-parallel β-barrel with four strands contributing from each monomer and flanked on either side by three α-helices. The overall structure of the LANA DBD is also similar to EBV EBNA1 and HPV E2 latent proteins (29–31). Recently, a multiple-point mutant kLANA protein crystal structure bound with kLBS1 fragment was reported and showed an asymmetric binding mode, different to the previously observed EBNA1 and E2 DNA complexes (18). Nine mutations were concentrated on the opposite side of the DNA binding interface to resemble the EBNA1 protein thereby improving the crystallizability of the LANA protein. In the DNA-free structures, crystal packing reveals both kLANA and mLANA DBD form higher oligomers, a closed ring with a bend occurring at the dimer–dimer interface in kLANA, while the mLANA forms linear oligomers in an end-to-end fashion (27–29). Disruption of this interface leads to impaired plasmid replication and maintenance *in vivo* (27,28). The biological relevance of the corresponding bend vs. linear tetramer formation displayed in the crystal structures of KSHV and MHV-68 LANA DBD still remains unclear due to the absence of any structural data in the presence of LBS1–2 TR DNA.

Here, we report that kLANA and mLANA are partially interchangeable in their function, as they can bind to the respective non-cognate LBS DNA *in vitro*. We show, however, that kLANA and mLANA bind DNA in solution very differently, validated by both isothermal titration calorimetry (ITC) thermodynamic profiling and small angle X-ray scattering (SAXS) low-resolution structural analysis. In addition, we show that the presence of tandem low- and high-affinity binding sites is obligatory for correct LANA binding. Higher oligomerization and its *in vivo* relevance to LANA function are discussed in detail with analysis of a new non-ring crystal form of kLANA-DBD and structure-based mutagenesis at the dimer–dimer interface.

## MATERIALS AND METHODS

### Gene cloning and expression

The region coding for KSHV LANA_DBD_ ((kLANA_1008–1150_); AAC55944.1) was amplified by PCR from an N- and C-terminal fusion LANA construct coding regions (kLANA_4–333:929–1162_). The regions were cloned into pET49b (+) vector (Novagen) using SmaI and HindIII restriction sites and includes a GST-6xHis-tag followed by a 3C protease cleavage site preceding the LANA protein sequence. The final protein sequence has six additional residues at the N-terminus after the removal of tag sequences by 3C protease. The resulting plasmids were transformed into *E. coli* BL21 Star (DE3) pRARE2 competent cells. ZYP-5052 auto-induction rich medium (32) supplemented with corresponding antibiotics kanamycin (100 μg/ml) and chloramphenicol (34 μg/ml) was inoculated with a pre-culture of these cells for expression. The cells expressing kLANA_1008–1150_ gene were kept under constant agitation at 37°C for 2 h followed by 20°C for 22 h of growth. Cells were then harvested by centrifugation at 4°C and the resulting pellets were either used immediately or frozen and stored at −20°C. Similar procedures were followed for producing various LANA_DBD_ constructs whose regions vary in the N- and C-terminal of DNA-binding domain (kLANA_1008–1150_; kLANA_1019–1150_; kLANA_1019–1162_).

In order to increase the stability of the kLANA_DBD_ protein and investigate the importance of tetramer formation, structure-based point mutations were performed in the kLANA_1008–1150_ construct using the QuikChange II XL Site-Directed Mutagenesis Kit (Agilent Technologies). Two groups of mutation were performed: at the dimer–dimer interface; residues Phe1037Thr and Ala1121Glu and at dorsal side of DBD protein (i.e. opposite-side to the DNA-binding interface); residues Lys1109Glu and Lys1140Glu. MHV-68 LANA_DBD_ ((mLANA_124–314_); NP_044913.1) was cloned into pET49b(+) as described previously (29) and the protein produced in a similar manner to kLANA_DBD_.

### Protein purification

For lysis 20 g of kLANA_1008–1150_ cell pellets were resuspended in ice-cold buffer A (25 mM Na/K Phosphate, 500 mM NaCl, 10% (v/v) glycerol, 1 M NDSB, pH 7.5) supplemented with 1 mg/ml lysozyme, 5 unit/ml OmniCleave (epicentre) and one complete EDTA-free protease inhibitor cocktail tablet (Roche) per 100 ml of resuspended cells, and incubated for 40 min at 4°C. Cells were lysed further using a Z Basic cell disruptor (Constant Systems Ltd). The lysate was cleared by centrifugation at 40 000 g for 40 min at 4°C. All the purification steps were performed using an ÄKTA Explorer 10 FPLC System at room temperature (GE Healthcare). The supernatant was passed through GSTPrep™ FF 16/10 column using a peristaltic P-1 pump at 4°C (GE Healthcare). The column was then washed with buffer A and eluted with buffer B (25 mM Na/K phosphate, 500 mM NaCl, 10% (v/v) glycerol, 20 mM glutathione, pH 7.5 (25°C)). The main fractions were pooled and 3C protease was added in a 1:100 molar ratio to GST-tagged kLANA protein with an addition of 2 mM DTT and 1 mM EDTA. The cleaved GST and unspecific DNA were removed from kLANA_DBD_ by further passing through HiPrep™ Heparin FF 16/10 column (GE Healthcare). The column was washed with buffer A and eluted using a linear gradient with buffer C (25 mM Na/K Phosphate, 2000 mM NaCl, pH 7.5 at 25°C). The protein was eluted around 1000 mM NaCl and the fractions were pooled, concentrated and injected into HiLoad™ 16/600 Superdex™ 75 pg column (GE Healthcare) which was pre equilibrated with buffer D (25 mM Na/K phosphate, 250 mM NaCl, 5% glycerol pH 7.0 at 25°C). Similarly kLANA mutant proteins and mLANA_124–314_ was also purified to its homogeneity.

### Crystallization and structure determination

Crystallization screening was performed for kLANA_1019–1150_ protein using a Cartesian MiniBee robot with the sitting-drop vapor-diffusion method in a sparse matrix screen. The crystallization drops were imaged regularly using Minstrel DT UV imager (Rigaku). Crystals of kLANA_1019–1150_ grew to full size within three days at 20ºC in 100 mM Citric acid pH 3.0, 1200 mM MgCl_2_ condition. Protein at 2.0 mg/ml concentration in buffer 10 mM HEPES, 1200 mM NaCl, pH7.5 was mixed in an equal ratio with precipitant. Cryoprotection was achieved by transferring the crystals to precipitant solution with the addition of 28% glycerol. X-ray diffraction data were collected at 100k at the European Synchrotron Radiation Facility, Grenoble, France. The data set were processed with *MOSFLM* and scaled using *SCALA* (33,34). The structure of kLANA_1019–1150_ was solved by molecular replacement using the program *PHASER* (35) with a dimer of kLANA DBD (PDB ID: 4K2J; (28)) as the search model. Subsequent model building and refinement was performed using *COOT* and *REFMAC5* (36,37). The atomic coordinates and structure factors are deposited in Protein Data Bank (PDB) with accession code 5A76.

### Protein–DNA modelling and structure analysis

kLANA and mLANA–DNA complex models for SAXS analysis were built by manual docking using PyMOL (v.1.5.0.4; Schrödinger). The kLBS1–2 and mLBS1–2 DNA models were prepared using 3D-DART web server (38). The protein interfaces and interaction surfaces were calculated and analyzed using the *PISA* webserver (39). Structural analysis was performed and figures prepared using *PyMOL*. Bend and rotation angles were calculated using the pymol draw_rotation_axis and angle_between_helices scripts, respectively.

### Small-angle X-ray scattering (SAXS)

The synchrotron radiation X-ray scattering data from solutions of free kLANA DBD, mLANA DBD, LBS1–2 DNA of KSHV and MHV-68 and from their complexes were collected on the P12 beamline of the EMBL on the PETRA-III storage ring (DESY, Hamburg, Germany) (40) and at the ESRF BM29 beamline (Grenoble, France) (41). Measurements at the P12 and BM29 beamlines were performed using PILATUS detector and automated filling (42). Data acquisition was performed at a sample-detector distance of 2.7 m, covering the range of momentum transfer 0.07 < *s* < 4.5 nm^−1^ (*s* = *4π sin*(*θ*)/*λ* where *2θ* is the scattering angle and *λ* = 0.12 nm is the X-ray wavelength) in 20 frames of 0.05 ms to check for possible radiation damage. The data were normalized to the intensity of the transmitted beam and radially averaged; the scattering of the buffer was subtracted and the difference curves were scaled for solute concentration. Primary data reduction was performed by automated pipeline (43) and comprehensive analysis of the scattering profiles was accomplished using the ATSAS software package (44). The forward scattering *I*(0) and the radii of gyration *R*_g_ were evaluated using the Guinier approximation, assuming that at very small angles (*s* < *1.3/R*_g_), the intensity is represented as *I*(*s*) = *I*(0) exp(−(*sR*_g_)^2^/3). The maximum dimension *D*_max_ was computed using the indirect transform package GNOM (45). Molecular weight (MW) estimate was made by comparison of the forward scattering with *I*(0) of bovine serum albumin (BSA) standard.

The scattering from the high resolution models was calculated using the program CRYSOL (46). Given the atomic coordinates, the program minimizes discrepancy in the fit to the experimental intensity *I*_exp_(*s*) by adjusting the excluded volume of the particle and the contrast of the hydration layer to minimize the discrepancy:

(1) \begin{equation*} \chi ^2 = \frac{1}{{N - 1}}\sum\limits_{j = 1}^N {\left[ {\frac{{I_{\exp } (s_j ) - cI_{{\rm calc}} (s_j )}}{{\sigma (s_j )}}} \right]^2 } , \end{equation*}

where *c* is a scaling factor, *N* is the number of points and *σ* denotes the experimental errors.

An ensemble optimization method (EOM) (47) has been applied to model the terminal portions of mLANA in solution acknowledging their flexibility. In the EOM approach, a large pool (of 10 000 models) with random conformations of the terminal loops has been generated and the optimized ensemble has been selected by genetic algorithm which compares the averaged theoretical scattering intensity fit to the experimental SAXS data. The distribution of the structural descriptors (i.e. *R*_g_, *D*_max_) in the optimized ensemble in respect to the original random pool provides an estimation of the conformation variability of macromolecule in solution.

The equilibrium mixtures were analyzed by the program OLIGOMER (48), which fits the experimental scattering intensity by a linear combination of the scattering profiles from its components. The individual intensities are weighted according to the volume fraction of the corresponding component of the mixture. The solution scattering data and models of LANA and LANA DNA complexes have been deposited in the small-angle scattering biological data bank with accession codes SASDAQ8, SASDAS8, SASDAP8, SASDAM8, SASDAR8 and SASDAN8.

### Preparation of oligonucleotides

**Figure 2.**
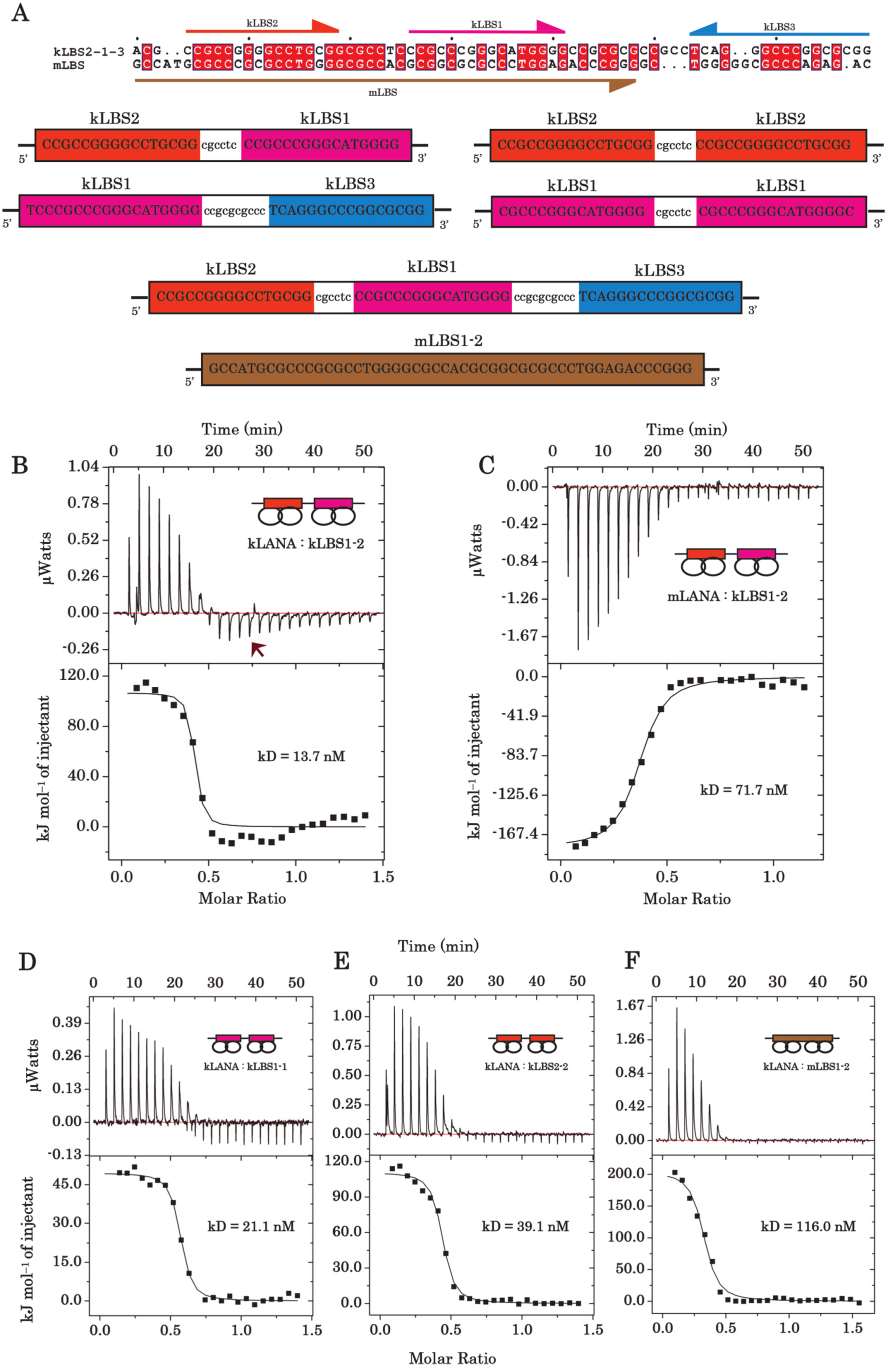
Analysis of kLBS1–2 binding by LANA. (**A**) Alignment and position of KSHV and MHV-68 LBS1–2 sequences, with kLBS1 (magenta), kLBS2 (orange), kLBS3 (dark blue) and mLBS1–2 (brown). The sequences that represent kLBS1–2, 1–3, 1–1, 2–2, 2–1–3 and mLBS1–2 DNA substrates are shown. Isothermal titration calorimetry of kLBS1–2 DNA titrated against (**B**) kLANA DBD and (**C**) mLANA DBD proteins. Contrasting energy was observed between kLANA and mLANA DBD by binding to kLBS1–2 DNA. The upper panel isotherms indicates the DNA binding raw data and shows the heat released versus time. The lower curve is obtained after integration of the peak intensities plotted against the DNA/protein molar ratio in the calorimeter cell. The solid lines correspond to the best fit using a one-site binding model. The arrow in panel A indicates a possible secondary binding event that occurs with kLBS1–2 binding. The contribution of high- and low-affinity sites to kLANA binding were analysed with (**D**) kLBS1–1, (**E**) kLBS2–2 and (**F**) non-cognate mLBS1–2 DNA were titrated against kLANA DBD protein. The thermodynamic parameters describing the fit for each LANA-DNA titration are presented in Table 1. Titrations were performed with a series of injections (25) of 1.5 μl of DNA to the protein samples.

All DNA oligonucleotides sequences (Figure 2A) were synthesized and purified using reverse-phase cartridge by Sigma–Aldrich. Oligonucleotides were dissolved in water at 2 mM stock solution and the complimentary strands (1:1 ratio) were annealed by heating at 95°C for 5 min (in a buffer containing 11.1 mM Tris pH 7.6, 55.5 mM NaCl, 5.5 mM MgCl_2_) and a step-wise decrease of 5°C over 2 h using MyCycler™ Thermal Cycler (BioRad). The annealed oligonucleotides were stored at −20°C prior to use.

### Isothermal titration calorimetry (ITC)

ITC titrations were performed with a MicroCal™ iTC200 Isothermal Titration Calorimeter (Malvern) at 25°C. Protein and DNA absorbance were measured after dialysis by NanoDrop (NanoDrop Technologies) and their concentrations were determined with their respective extinction coefficients. Twenty-five 1.5 μl injections of each DNA were titrated into LANA_DBD_ protein solution. Data were corrected for nonspecific heats and analysed using MicroCal Origin^®^ 7.0 software using a one-site binding model. CHASM software was used for fitting and calculating thermodynamics parameters for a two-site binding model (49). The experiments were performed in triplicate and showed similar results. Proteins were freshly prepared for the ITC experiments and used within 24 h after the purification from the gel-filtration column, which is crucial due to the kLANA DBD's propensity to form higher oligomers. The annealed dsDNA oligonucleotides were dialysed overnight at 4°C using Slide-A-Lyzer^®^ Mini dialysis unit (Thermo Scientific) against buffer D (25 mM Na/K phosphate, 250 mM NaCl, 5% glycerol pH 7.0 (25°C)).

## RESULTS

### Oligomerization and solubility properties of LANA DBD

The kLANA, mutant kLANA and mLANA DBD proteins are all able to form a stable dimer as confirmed by gel-filtration chromatography (Supplementary Figure S1). We show that LANA DBDs also form tetramers and subsequently higher-oligomers, this depends on both the protein and salt concentration. The kLANA DBD has a greater propensity to form higher oligomers than the mLANA DBD proteins (Supplementary Figure S1) kLANA_1019–1150_ was eluted as a dimer in the presence of 1 M NaCl and was used for crystallization and structural studies. We and others have observed that the kLANA DBD protein truncations can be purified either by refolding or by maintaining high, up to 1M NaCl concentrations (27,28). However, such high salt concentration hampers its biophysical analysis. In an attempt to increase solubility without interfering with LANA DBD's DNA-binding and oligomerization properties, we have made structure-based point mutations in the positively charged surface at the dorsal side of kLANA (opposite-side to the DNA-binding face) (Supplementary Figure S1C). The dorsal side of LANA DBD was shown to be important in interacting with the chromosome associated bromodomain proteins (BRD-2 and BRD-4) (27–29). Lys1109 and Lys1140 residues which are present at the start of α-helix 3 and end of β-strand 4, respectively, were independently mutated to a negatively charged glutamate residue in the kLANA_1008–1150_ construct (see Supplementary Figure S1C). Lys1109Glu but not Lys1140Glu greatly increased the solubility of the protein and we achieved concentrations greater than 2 mg/ml with a minimum of 250 mM NaCl. The kLANA_1008–1150 (K1109E)_ protein was predominantly a dimer, with a small proportion present as a tetramer, which increased with extended storage at 4°C. All the following thermodynamics studies were performed with freshly purified kLANA_1008–1150 (K1109E)_ protein.

### ITC analysis

LANA DBD proteins were shown by gel-shift assay, to bind to their LBS sites within the KSHV and MHV-68 TR DNA (20,29,50–52). Each terminal repeat sequence consists of two consecutive high (LBS1) and low (LBS2) affinity sites (Figure 2A). Both sites individually or joined together (LBS1–2) were able to form a complex with LANA as shown using gel-shift assay (15,29). But so far the mode in which LANA binds to the LBS DNA and their thermodynamics have not been studied. Also given the high structural similarities between KSHV and MHV-68 LANA DBD, it is interesting to probe whether these proteins can be interchanged to bind to their homologous non cognate TR DNAs. Using ITC, thermodynamics parameters including ΔG (Gibbs free energy), ΔH (enthalpy), −*T*Δ*S* (entropy), *k*_D_ (dissociation constant) and *n*-value (number of binding sites) were determined at 25ºC.

#### KSHV and MHV-68 LANA-DBD display different thermodynamic binding profiles

First, the kLBS1–2 DNA was titrated against the kLANA DBD and shows binding governed by endothermic energy (Figure 2B). kLANA–kLBS1–2 binding signature has Δ*H* = 101.4 kJ/mol and −*T*Δ*S* = −146.3 kJ/mol with a *k*_D_ of 13.7 nM. The favourable ΔS indicates the combination of binding events: desolvation at the kLANA–DNA interface and distortion of DNA. Titration of non-cognate kLBS1–2 DNA against the mLANA DBD released heat indicating that cross-binding is exothermic (Figure 2C), similar to its cognate mLBS DNA binding (29). The mLANA–kLBS1–2 binding signature has Δ*H* = −173.7 kJ/mol and −*T*Δ*S* = 132.8 kJ/mol with a *k*_D_ of 71.7 nM, which is 5-fold lower than kLANA–kLBS1–2 binding (see Table 1). The favourable Δ*H* indicates that unlike kLANA, mLANA binding is driven by hydrogen-bonding and van der Waals interactions with the DNA. Although the binding mode for KSHV and MHV-68 LANA to kLBS1–2 sites differ, the gained Gibbs free energy of binding are similar: Δ*G* = −44.9 kJ/mol and −40.1 kJ/mol for kLANA and mLANA, respectively. Hence, the binding of both proteins with kLBS1–2 are spontaneous processes. The Δ*H* and −*T*Δ*S* values depend on the experimental conditions, such as salt, pH, and temperature, however, in the same condition two homologous proteins obtaining contrasting binding modes with one DNA is remarkable.

**Table 1.** Thermodynamic parameters for binding of LANA to LBS1–2 TR DNA

Sample		Δ*G* (kJ/mol)	Δ*H* (kJ/mol)	(−)*T*Δ*S* (kJ/mol)	*k*_D_ nM	*n*	Relative percentage
Syringe	Cell						
kLBS1–2	kLANA	−44.9	101.4	−146.3	13.7	0.40	100
	mLANA	−40.9	−173.7	132.8	71.7	0.35	19
kLBS1–1	kLANA	−43.9	47.4	−91.3	21.1	0.54	65
kLBS2–2		−42.2	111.4	−153.6	39.1	0.41	35
mLBS1–2		−39.6	211.0	−250.6	116.0	0.29	12
kLBS1–2	kLANAakM	−34.5	119.0	−153.5	890.0	0.43	2

#### Both LBS1 and LBS2 are required for effective binding to kLANA-DBD

To access the importance of sequence and arrangement of LBS1 (high-) and LBS2 (low-affinity) present within the TR region of the viral genome, we have assembled DNA sequences kLBS1–1, kLBS2–2 and mLBS1–2 (Figure 2A) and determined the binding affinity with kLANA DBD protein (Figure 2D-F). In comparison to the kLANA DBD kLBS1–2 complex, the three hybrid DNAs were also able to bind to kLANA DBD with a similar endothermic binding mode and with each DNA ligand at least two dimer molecules were calculated to bind. However, compared with kLANA–kLBS1–2 complex, kLANA's binding affinities decreased modestly with 1.5-fold for kLBS1–1 and 3-fold for kLBS2–2 (Figure 2D and E) but still in the low nanomolar range. Interestingly, the presence of two consecutive high-affinity sites did not increase the binding affinity to kLANA-DBD (see Table 1). While the gained Gibbs free energy are similar for the various LANA-DBD LBS complexes there was significant differences in their enthalpy-entropy contributions (see Table 1). The kLANA-DBD also bound non-cognate mLBS1–2 sites endothermically, similar to kLBS1–2 binding, but with an 8-fold lower affinity (Table 1).

It is interesting to note that kLANA and mLANA DBD were able to bind non-cognate DNA in lower μM to nM range, even though there is very little DNA sequence conservation between the two (Figure 2A).

### Structure of kLANA DBD in a non-ring conformation illustrates the flexibility of its tetramer interface

**Figure 3.**
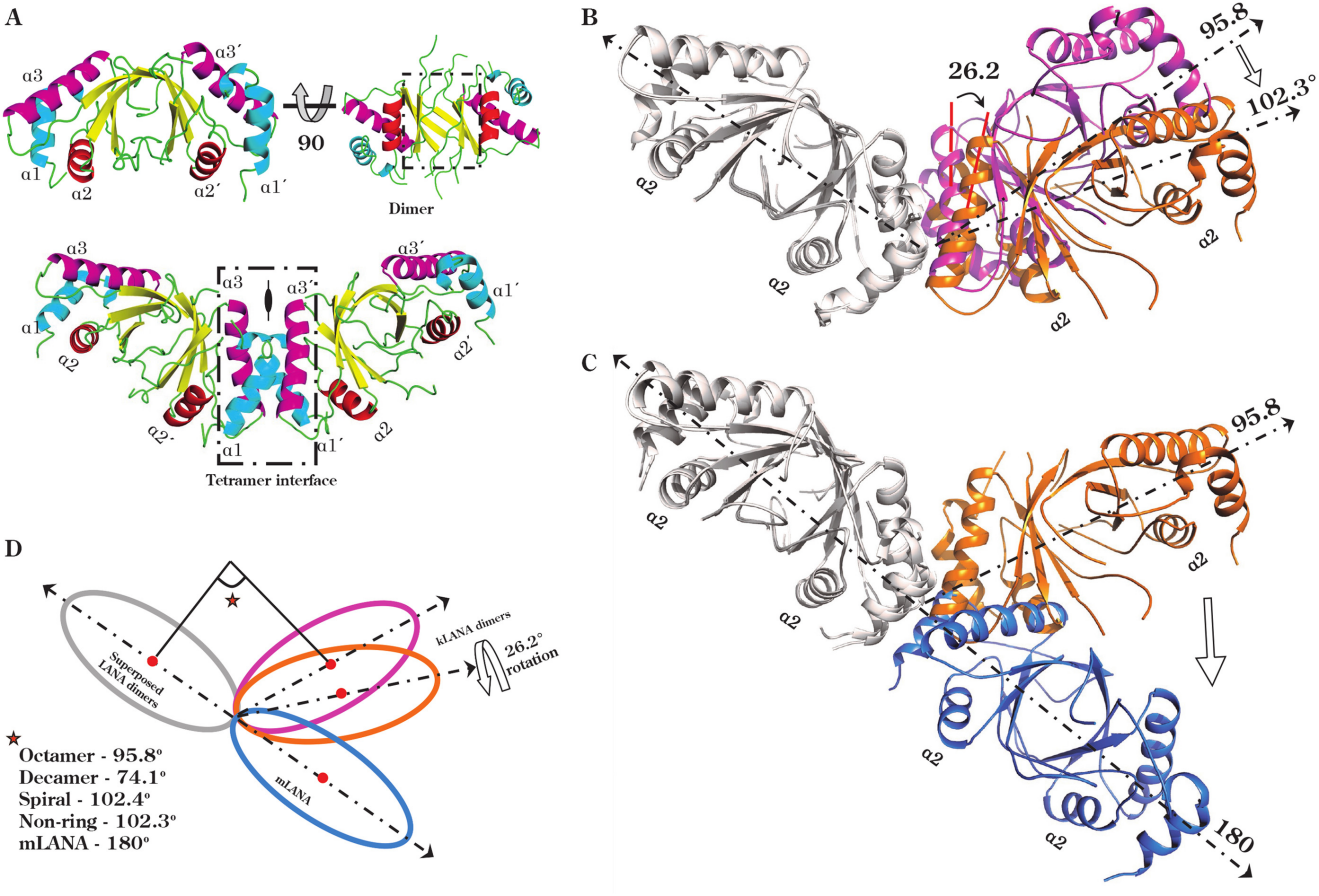
kLANA non-ring conformer exhibits a dimer–dimer twist. (**A**) kLANA dimer structure shown in two orientations (upper panel) and the tetramer assembly (lower panel). The helix α2 colored in red is used for DNA binding and helices α1 and α3 that are involved in the tetramer interface are colored in cyan and pink, respectively. The bent tetramer interface is formed at the 2-fold axis. Dimer and tetramer interfaces are boxed by dotted lines. (**B** and **C**) Cartoon illustration of the different bend angles observed at the tetramer assembly interface. Dimer 1 of both tetramers are colored in gray which were superposed to show the differences in dimer 2 orientation. (**B**) Octamer ring structure (magenta; 2YQ0) bends 96° and while non-ring structure (orange; 5A76) bends 102° with an additional 26° rotation (**C**) mLANA tetramer (light blue; 4BLG) forms a linear conformation. (**D**) Schematic representation of each dimer represented as spheres. The bend angels at the tetramer assembly interfaces varies between 74° and 102° in the four different kLANA DBD crystal forms, only two of which are shown for clarity (2YQ0, colored magenta, octamer ring bend angle of 96°; 2YPY, decamer ring bend angle of 74°; 4UZC, spiral bend angle of 102° and 5A76, colored orange, non-ring bend angle of 102°). mLANA linear tetramer formation (4BLG, colored light blue, bend angle 180°).

Two groups have previously reported the crystal structure of kLANA DBD forming a stable dimer structure similar to EBNA1 and mLANA; however, in the crystal structures only the kLANA DBD forms a higher order ring structure with four or five dimer molecules or an infinitive number of dimer molecules forming a spiral arrangement (18,27–28). Whether these higher order kLANA DBD ring structures exist *in vivo* is unclear. Here we report the crystal structure of kLANA DBD forming a novel non-ring structure in the crystal packing (Supplementary Figure S2). The structure was resolved at 3.8 Å resolution in space group C2221 (Figure 3A). The kLANA DBD structure determined consists of amino acid residues 1019–1150 with an additional two amino acid residues glycine and proline at the N-terminus resulting from the 3C protease tag cleavage. The asymmetric unit consists of four dimer molecules (Supplementary Figure S2). The structure is of very good quality as indicated by a MolProbity score of 1.85 (100th percentile (*n* = 342)) (53), see Table 2.

**Table 2.** Data collection and refinement statistics

X-ray diffraction data	
Wavelength (Å)	0.9725
Space group	C2221
Cell dimensions: *a*, *b*, *c* (Å)	200.2, 200.2, 83.6
*α*, *β*, *γ* (°)	90.0, 90.0, 90.0
Resolution (Å)	47.2–3.8 (4.25–3.80)
*R*_merge_^a^	0.279 (0.58)
*R*_pim_^b^	0.107 (0.22)
*I*/*σI*	5.2 (3.2)
Completeness (%)	99.0 (99.2)
Redundancy	7.5 (7.8)
Total measured reflections	125 845 (36 404)
Unique reflections	16 786 (4 686)
Wilson *B*-factor (Å^2^)	50.2
Refinement	
Resolution (Å)	141.6–3.8
*R*_cryst_/*R*_free_^c^	0.22/0.27
Protein atoms	7 336
*B* factor (Å^2^)	80.7
RMSD bond length (Å)	0.012
RMSD bond angles (°)	1.384
Ramachandran analysis: favored/allowed (%)	97.6/2.4

Values in parentheses are for the highest resolution shell.

^a^$R_{merge} = \sum\nolimits_{hkl} {\sum\nolimits_i {|I(hkl)_i - \langle I(hkl)\rangle |} } /\sum\nolimits_{hkl} {\sum\nolimits_i {I(hkl)_i } }$,

^b^$R_{pim} = \sum\nolimits_{hkl} {\sqrt {1/n - 1} \sum\nolimits_i {|I(hkl)_i - \langle I(hkl)\rangle |} } /\sum\nolimits_{hkl} {\sum\nolimits_i {I(hkl)_i } }$, where *I*(*hkl*) is the intensity of reflection *hkl* and 〈*I*(*hkl*)〉 is the average intensity over all equivalent reflections.

^c^$R_{cryst} = \sum\nolimits_{hkl} {|F_o (hkl) - F_c (hkl)|} /\sum\nolimits_{hkl} {F_o (hkl)}$. *R*_free_ was calculated for a test set of reflections (5%) omitted from the refinement.

The structure of kLANA DBD belongs to α–β family with a topology of α–β plaits. The central two anti-parallel β-sheets of two monomers form a β-barrel dimer by burying several hydrophobic residues with an average buried surface area of 1123 Å^2^ per monomer (Figure 3A). The three helices α1, α2 and α3 from each monomer flank the core β-barrel structure, α1 and α3 is involved in the higher oligomerization (see below) and α2 is proposed to interact with the DNA major groove (51,52). The overall monomer and dimer structure of kLANA DBD is similar to the previously reported kLANA structures with an rms deviation of ∼0.8 Å and to homologs MHV-68 LANA (1.6 Å) and EBNA1 (2.4 Å) for the equivalent residues from dimer molecules. kLANA DBD also forms a tetramer by using helices α1 and α3, burying an average surface area of 545 Å^2^ per monomer (Figure 3A). The tetramer interface forms a bend angle of about 102° between the two dimer molecules and is related by a crystallographic two fold axis (Figure 3A). A similar interface has been reported previously but the angle between the dimer–dimer interfaces are 96° and 74° (the corresponding reported bend angles were 90° and 72° (27)) forming octameric and decameric ring structure, respectively (Figure 3B and D and Supplementary Figure S7).

In addition to the bend angles, a greater rotational angle between dimers has been observed in the present structure, indicating more flexibility at this interface than previously known (Figures 2D and 3B). The relative dimer rotation between four different crystal structures (octamer ring, decamer ring, spiral and non-ring) was calculated by superpositioning dimer1 of all tetramers and measuring the angle between the respective helix α3 of dimer2. A maximum rotation angle of ∼34° was observed between helix α3 of the non-ring and decameric ring structures. With respect to the non-ring crystal structure, the relative rotation angles were 26° (octamer) and 14° (spiral) (Supplementary Figure S8 and Table S1). This ability to rotate may facilitate kLANA's cooperative binding and bending of DNA. The superposition of kLANA with mLANA shows a larger movement of dimer 2 (Figure 3C and D).

### SAXS analysis

We wanted to clarify the conformational differences observed in dimer–dimer assembly within kLANA and mLANA DBD crystal structures using SAXS. This technique gives information on the shape and oligomerization of proteins in solution and provides structural characterisation of the disordered regions missing in high-resolution crystal structures.

#### kLANA and mLANA DBD oligomeric assembly

**Figure 4.**
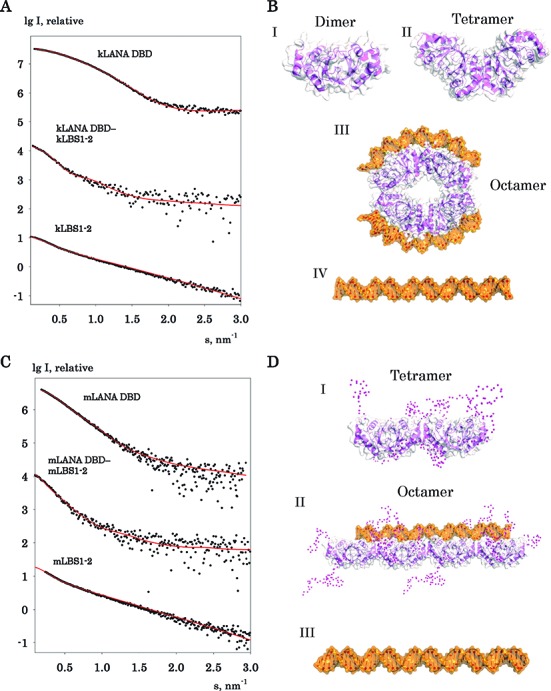
SAXS modelling of LANA and LBS1–2 DNA complexes. The scattering profiles of kLANA and mLANA and with its complexes. (**A** and **B**) The left-side panels show experimental SAXS scattering curves (black dots). The fits corresponding to the various models are shown in red solid lines. The experimental scattering curves agree well with the theoretical scattering curves (red). The right-side panels show the best fitted models as a surface representation. (**A**) The scattering of the kLANA DBD sample represents a population composed of both dimer (I) and bent tetramer (II). The kLANA DBD LBS1–2 sample represents an octameric complex with each tetramer bound to a kLBS1–2 DNA (III) with a small percentage of free tetramer (II) and DNA (IV). The scattering of the kLBS1–2 DNA sample alone represents a linear conformation (IV). (**B**) The scattering mLANA DBD maintains a linear tetramer conformation (I) and linear octamer in the presence of mLBS1–2 DNA (II). Similarly, the scattering of the mLBS1–2 DNA sample alone represents a linear conformation (III). Experimental fitting of polydisperse data used the OLIGOMER program, while all other monodisperse data were fitted with the program CRYSOL. N- and C-terminal residues (magenta spheres) not present in the mLANA model were generated by the EOM program (panel D I and II).

SAXS analysis showed that kLANA_1008–1150_ and kLANA_1019–1162_ proteins were present in a polydispersed state and exhibited a strong concentration-dependent equilibrium between dimer, tetramer and higher oligomers. Due to the polydispersed state accompanied with large oligomers, none of the individual models (dimer, three different bent tetramers, octamers, decamers and linear tetramer model based on mLANA) were able to convincingly fit the scattering curve. However, the dorsal mutant kLANA_1008–1150 (K1109E)_ protein behaved somewhat better and SAXS data at *c* = 2.5 mg/ml indicated that up to 80% of species present in solution as a dimer and 20% in the form of the bent tetramer yielding a *χ*^2^ of 0.83 by the program Oligomer (see Figure 4A and B I and II) (note: the mutated residue is not in the locality of the oligomerization sites; see above). Unlike kLANA DBD, the mLANA_124–314_ protein displayed no concentration-dependent oligomerization and was present as a linear tetramer. The missing N- and C-terminal residues from the crystal structures were built using ensemble optimization method (EOM) program and the selected EOM models yielded a fit to the scattering curve with *χ*^2^ 1.70 (see Figure 4C and D I). The built N- and C-terminal residues by EOM are likely to be disordered and protruding out of the core structure (Figure 4D I). In summary, similar to the observed crystal structures, kLANA favors the bent tetramer conformation, while mLANA stays in a linear conformation in solution.

#### Solution states of the kLANA and mLANA DBD–DNA complexes

Since there is no structure of LANA bound to LBS1–2 TR DNA, we employed SAXS to determine the overall shape of the complex and to determine if there is any structural change with LANA upon binding to DNA. For the LANA complexes, we used the respective KSHV and MHV-68 cognate LBS1–2 DNA. Models of kLBS1–2, mLBS1–2 and LANA-DNA complexes were built manually with the help of different LANA and EBNA1–DNA complex structures (for details see Materials and Methods section).

First, we measured the LBS1–2 DNA of KSHV and MHV-68; both were elongated in shape and correlated well with the model with *χ*^2^ 2.9 and 1.5, respectively (see Figure 4A; B IV and C; D III). Addition of kLBS1–2 DNA to kLANA_1008–1150_ greatly increased the solubility of the protein up to a concentration of 5mg/ml at 250 mM NaCl. However, we again observed a strong concentration-dependent aggregation with the kLANA-kLBS1–2 complex; only at lower concentrations the polydispersity of the samples reduced with almost no observed aggregation. Using PRIMUS and GNOM, the radius of gyration (*R*_g_) 48 Å and the maximum dimension (*D*_max_) of 160 Å were obtained from the scattering curve of the kLANA–kLBS1–2 complex, which were larger than the parameters obtained for free kLANA_1008–1150_ or kLBS1–2 DNA indicating complex formation (see Table 3). By using the Oligomer program, we found that the major component of the mixture (74%) could be assigned to kLANA–kLBS1–2 species present in an octameric state with two bound kLBS1–2 DNA as presented in Figure 4A and B III (see also Materials and Methods section). Equimolar amounts of free tetramer and kLBS1–2 DNA were selected as the other components of the mixture to fit the polydisperse scattering curve with a *χ*^2^ value of 1.06 (Figure 4B II and IV). Of note: the observation of an octamer ring structure in solution of kLANA when bound to DNA has been observed in one of the DNA-free crystal structures, however, the *in vivo* relevance of the octamer is still not clear. Neither the linear nor bent tetramers bound to DNA as a single component were able to completely satisfy the scattering curves. In the case of mLANA_124–314_, the addition of mLBS1–2 DNA again gave larger *R*_g_ (58 Å) and *D*_max_ (200 Å) values compared to protein or DNA alone. A tentative octamer model was built from two copies of an EOM tetramer model of mLANA protein positioned side by side and one mLBS1–2 DNA bound in the middle of the octamer. The mLBS1–2 contains 50bp compared to 46bp for kLBS1–2; however, the precise position of LBS1 and LBS2 sites within the mLBS1–2 DNA is still ambiguous. It is possible that the mLBS1–2 50 bp includes an additional binding site to accommodate a second tetramer (Figure 4D II). Nevertheless, mLANA-mLBS1–2 complex modelled as a linear octamer yielded a very good fit to the scattering curve to a *χ*^2^ value of 1.01 (see Figure 4C and D II) and more importantly no bending of DNA has been observed.

**Table 3.** SAXS data collection and statistics of LANA and LANA-DNA complexes

Sample	kLANA _1008–1150 Lys1109Glu_	kLANA _1008–1150_ –kLBS1–2	kLBS1–2 _(38 bp)_	mLANA _124–316_	mLANA _124–316_ – mLBS1–2	mLBS1–2 _(50 bp)_
**Data collection parameters**						
Beam line	BM29	P12	BM29	P12	P12	P12
Beam geometry (mm^2^)	0.7×0.7	0.2×0.12	0.7×0.7	0.2×0.12	0.2×0.12	0.2×0.12
Wavelength (nm)	0.09	0.124	0.09	0.124	0.124	0.124
s range (nm^−1^)	0.03–5.0	0.07–4.5	0.03–5.0	0.07–4.5	0.07–4.5	0.07–4.5
Exposure time (s)	1	1	1	1	1	1
Concentration range (mg/ml)	0.5–2.5	0.4–6.3	0.13–0.50	0.6–5.0	0.6–2.5	1.2–5.0
Temperature (K)	293	283	293	283	283	283

**Structural parameters**						
*R*_g_ (nm) [from Guinier]	2.4±0.1	4.8±0.1	4.0±0.1	4.2±0.1	5.8±0.1	4.0±0.1
*R*_g_ (nm) [from P(r)]	2.4±0.1	4.8±0.1	4.1±0.1	4.4±0.1	6.0±0.1	4.5±0.1
*D*_max_ (nm)	9.5±0.5	16±0.5	16±0.5	16±0.5	20±0.5	16±0.5
Porod volume estimate (nm^3^)	50	250	50	170	475	50
SAXS accession code	SASDAQ8	SASDAS8	SASDAP8	SASDAM8	SASDAR8	SASDAN8

The observed kLANA bent and mLANA linear tetramer conformations were unaffected by binding to cognate LBS1–2 DNAs. Instead, binding to DNA induces higher oligomerization up to octamer in both kLANA and mLANA which might reflect the need of oligomerization for *in vivo* function. Notably, kLANA and mLANA distribute similarly in cells (Supplementary Figure S6), both in the presence or absence of episomes, consistent with their common function.

### Pivot mutation at the tetramer interface reduces the kLANA–DBD binding to kLBS1–2

**Figure 5.**
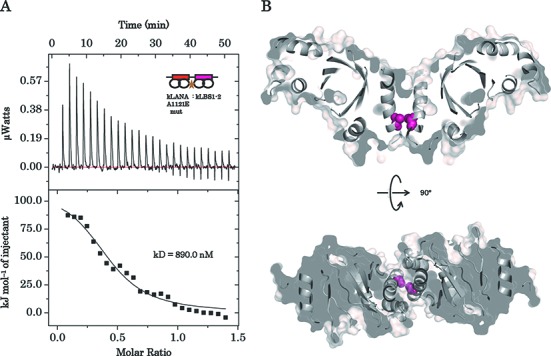
The effect of a kLANA pivot mutation on kLBS1–2 DNA binding. Isothermal titration calorimetry of kLBS1–2 DNA titrated against the (**A**) tetramer interface mutant, residue 1121 alanine is mutated to glutamate. (**B**) Cross section of the tetramer interface in two different orthogonal views, Ala1121 (pink sphere) is present at the pinnacle of the pivot interface.

kLANA-DBD protein forms a strong dimer (54), our crystal structure and those recently reported have identified the regions necessary to form a tetramer (27,28). Tetramer formation is important for binding to the consecutive high- and low-affinity LBS DNA. Although the tetramer interface is flexible (see above), residue alanine 1121 at the C-terminal end of α-helix 3 is in close contact with the equivalent residue of dimer 2 at the tetramer interface to form a pivot (Figure 5B). The Cα–Cα distance between the respective alanine 1121 residues is ∼5.0–6.0 Å in all three kLANA–DBD crystal structures, the close proximity has highlighted the importance of this residue in the oligomerization interface. To probe further the importance of the bending at the tetramer interface for DNA binding we mutated the alanine 1121 to a negatively charged glutamate residue to disrupt this close proximity pivot point. The Ala1121Glu mutation has also been shown to greatly hamper the replication and speckle formation in KSHV infected cells (18). The Ala1121Glu mutation was introduced into the kLANA_1008–1150 (K1109E)_ mutant construct resulting in the double mutant kLANA_k1109E, A1121E_. The double mutant protein was produced in a similar manner to other proteins but showed less tetramer species by size exclusion analysis (Supplementary Figure S1). kLANA_k1109E, A1121E_ was titrated against kLBS1–2 DNA, but failed to produce measurable heat upon binding in the ITC experiment at 25°C (Supplementary Figure S3) even when using higher protein and DNA concentrations. However, reducing the experiment setup from 25 to 4°C allowed measurement of binding heat and calculation of thermodynamic values. The tetramer interface Ala1121Glu mutant bound kLBS1–2 governed by endothermic heat, but with a 65-fold reduction in affinity (Figure 5A and Table 1). The thermodynamics values were Δ*H* = 119 kJ/mol and −*T*Δ*S* = −153 kJ/mol and Δ*G* = −34.5 kJ/mol with a *k*_D_ of 0.8 μM. The *n*-value (0.4) remained similar to wild-type complex, i.e. two dimer molecules are calculated to bind per kLBS1–2 DNA. The mutation at the tetramer interface was expected to hinder the tetramer formation resulting to increase in the *n*-value up to 1, i.e. one dimer molecule to bind per kLBS1–2 DNA. Surprisingly the *n*-value remained unchanged indicating the mutation did not abolish the tetramer formation but likely to alter its conformational degree of freedom needed for cooperativity, hence its reduced affinity.

### kLANA exhibits a biphasic binding mode with kLBS2–1–3

**Figure 6.**
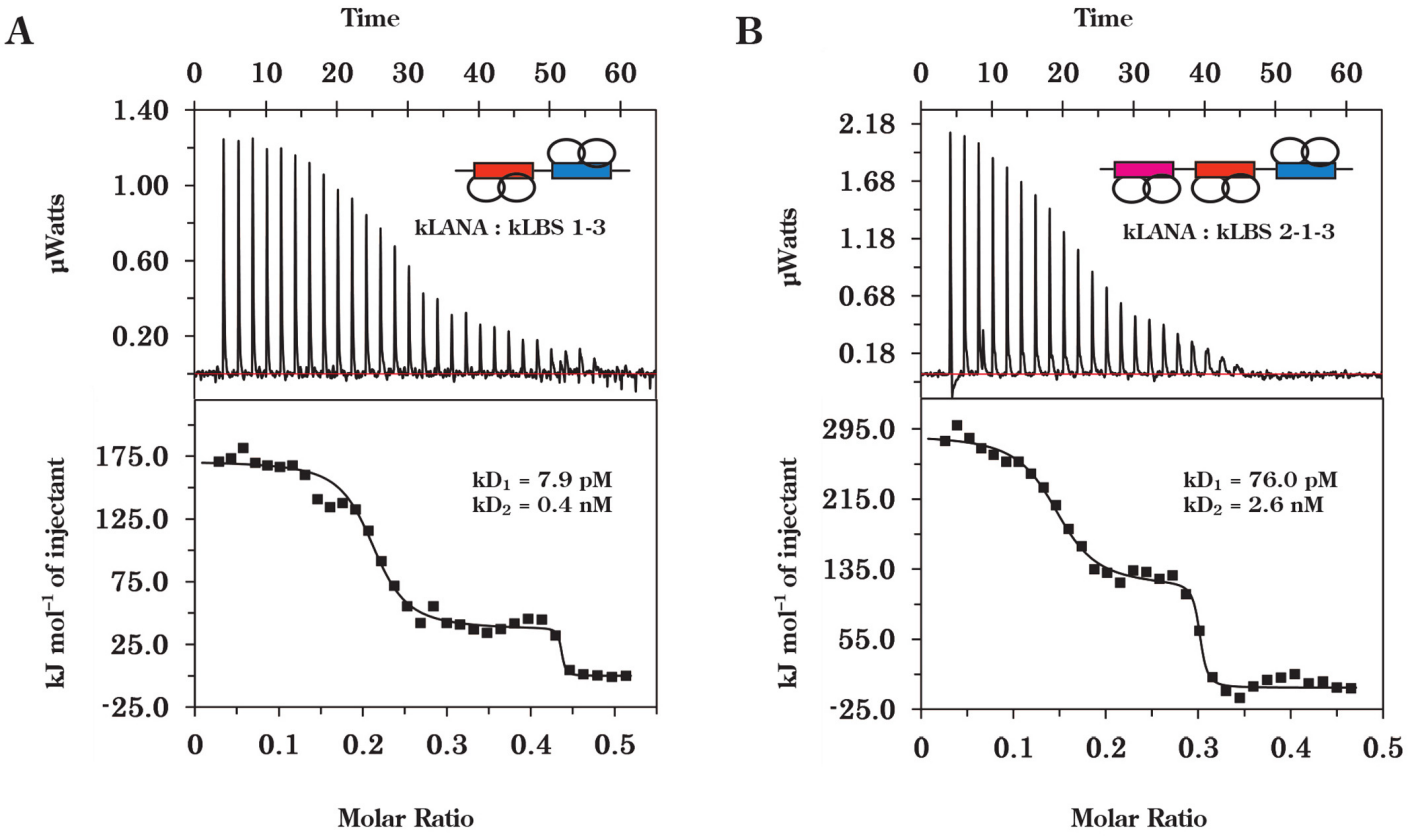
LBS3 induces biphasic binding by kLANA. ITC analysis of the binding of kLANA to kLBS1–3 (**A**) and kLBS2–1–3 (**B**). A biphasic binding profile was observed for both DNA substrates (top panel), which was fitted using a two-site binding model (bottom panel). The thermodynamic parameters describing the fit are presented in Table 4.

In addition to the LBS1–2, a third LBS (LBS3) was recently identified to be located in the opposite-strand adjacent to LBS1 (Figure 2A) (18). Earlier, Hu and Renne have termed the region associated with LBS3 as a replication element (55). Similar to LBS2, LBS3 is a low-affinity site and has a lower μM affinity to kLANA (18). We have now identified that the addition of LBS3 with LBS1 and LBS1–2 increased the binding affinity for kLANA and alters the binding profile from single to a biphasic. However, the binding events, analogous to other kLANA-kLBS interactions, are governed by endothermic energy (Figure 6). Even though kLANA binding to LBS2 and LBS3 are similar (18), when combined with LBS1 the binding profile changes from single to biphasic for LBS3. The differences could be attributed to the longer spacer region between LBS1 and LBS3 (Figure 2A). The binding affinities and thermodynamics parameters were calculated by fitting to a two-site binding mode (Table 4).

**Table 4.** Thermodynamic parameters for binding of kLANA to kLBS1–3 and kLBS2–1–3

Sample		Δ*G*_1_ (kJ/mol)	Δ*G*_2_ (kJ/mol)	Δ*H*_1_ (kJ/mol)	Δ*H*_2_ (kJ/mol)	(−)*T*Δ*S*_1_ (kJ/mol)	(−)*T*Δ*S*_2_ (kJ/mol)	*k*_D1_	*k*_D2_
Syringe	cell								
kLBS1–3	kLANA	−63.5	−53.7	179.0	39.1	−242.0	−92.8	7.2 pM	0.39 nM
kLBS2–1–3		−57.8	−48.9	293.0	108.0	−350.0	−156.0	74.2 pM	2.68 nM

### kLANA and mLANA redistribute to nuclear dots in the presence of episomal DNA

We assessed LANA's distribution in cells in the absence or presence of viral episomal DNA, which contains the LANA binding sites within TR elements. Consistent with previous descriptions (20,56), kLANA (green) broadly distributed throughout the nuclei (red) of uninfected BJAB B lymphoma cells (Supplementary Figure S6A and S6B) (overlay of green and red generates yellow), but concentrated to dots in the presence of k8TR episomal DNA (Supplementary Figure S6C and S6D). Similarly, mLANA (green) distributed broadly throughout the nuclei (red) of MEF cells (Supplementary Figure S6E and S6F), (overlay of green and red generates yellow), but concentrated to dots in the presence of m4TR episomes DNA (Supplementary Figure S6G and S6H). Each LANA dot indicates a viral episome (20).

## DISCUSSION

How LANA assembles and executes its multiple functions in the herpesvirus life cycle has remained enigmatic. We and others have shown that the LANA functional unit is not a monomer, but is instead dimeric and it is the building block to its multiple functions. In previous crystal structures, kLANA DBD forms octameric, decameric ring, or a spiral structural assembly while mLANA forms linear oligomers connected in an end-to-end fashion (18,27–29). Here we report another crystal form of kLANA DBD which forms similar dimer and bent tetramer assemblies despite not showing a ring or a spiral structure using these two interfaces. Two distinct oligomerization interfaces are present, first, the dimer-interface made by two antiparallel β-sheets from two monomers to form a thermostable dimer, which is conserved in the origin binding proteins (OBP) from herpesviruses (LANA and EBNA1) and papillomavirus (E2). Secondly, the assembly-interface, where two dimers are expected to form a tetramer using the flanking helices α1 and α3 in herpesvirus OBP proteins (27–28,30,57). This assembly interface is more variable within OBPs. For example, the helix α1 is absent in papillomavirus E2 dimer protein and lacks the ability to form higher oligomers (58). We show here that in solution, similar to the crystal structures, kLANA formed dimer or bent tetramer while mLANA formed a linear tetramer. It is possible that LANA can alter its shape, through oligomerization, to create different assemblies: a *bat-wing* shape composed of two dimers (kLANA), a linear assembly of two or four (mLANA), a ring formed by four or five assembled kLANA dimer molecules and an infinitive number of dimers joined to form a spiral conformation. These assemblies may each have designated functions for both KSHV and MHV-68; for example, the viral matrix protein, VP40 has evolved to coordinate different functions in the Ebola viral-life cycle through its multiple oligomeric assemblies (59).

Since all the OBPs have a similar dimer structure, both mLANA and kLANA DBDs are expected to use the α2 helix to recognize DNA sequences in the major groove (18,29,51–52). Interestingly, kLANA and mLANA have different modes of binding to LBS1–2 DNA (Figure 3). The kLANA DBD binding to kLBS1–2 DNA is endothermic. Similarly, binding to non-cognate DNA, i.e., kLANA-mLBS1–2 DNA is also endothermic, albeit of weaker affinity. In contrast, mLANA binds both cognate (29) and non-cognate kLBS1–2 DNA exothermically. Thus, our data indicates that it is LANA, not the DNA species that determines the binding mode.

We can deduce from previous ITC studies on protein–DNA interactions (60,61), that the favourable enthalpy observed in mLANA binding implies greater major groove helix α2 contributions, while kLANA less so. This is also supported by the kLANA LBS1 DNA structure, where only one helix α2 per dimer interact with the major groove (18). The LBS1 and LBS2 sites are each expected to bind one LANA dimer, thus formation of tetramer is required for binding with LBS1–2 DNA. In agreement with this hypothesis, we obtained by ITC a stoichiometric n-value, close to 0.5 (Table 1). Furthermore, our SAXS measurements (Table 3) show that in solution, kLANA maintains a bent conformation when bound to LBS1–2, but additionally assembles to an octamer ring with two LBS1–2 bound (*R*_g_ = 4.8) (see Figure 4A and B III). mLANA also forms a octamer but remains linear with LBS1–2 DNA bound (*R*_g_ = 5.8) (Figure 4C and D II). Collectively, our data suggests that the contrasting binding energy observed in ITC reflects kLANA bending the DNA upon binding, while mLANA binds DNA in a linear fashion.

In addition, supporting evidence comes from Jacobson et al., who have shown a striking relationship between the degree of imposed DNA distortion and the thermodynamics parameters of each system (60). For complexes with relatively undistorted DNA, such as GCN4 transcription factor and λcl repressor binding to a symmetric operator DNA, favourable Δ*H* change drives unfavourable Δ*S* change, whereas for complexes with highly distorted DNA such as CAP protein and TATA box binding protein, unfavourable Δ*H* is driven by favourable ΔS (62–64). In agreement, Wong and Wilson have shown using circular permutation assay that kLANA bends kLBS1–2 DNA by about 110° (65).

Regardless of the binding modes, both kLANA and mLANA bind to reciprocal DNA, this fuels the idea that it might be feasible to substitute mLANA with kLANA within MHV-68 virus and to explore kLANA function in a mouse model of infection. We also show that the presence of consecutive high- and low-affinity sites in LBS1–2 is essential for proper binding to kLANA DBD. Replacing the low-affinity site with another high-affinity site to form LBS1–1 did not improve the binding affinity of kLANA (Figure 2D-F and Table 1). In agreement with this result, there is no functional enhancement of episomal replication by LANA acting on LBS1–1 instead of LBS1–2 DNA (15). Since the replication element, now identified as LBS3, is left untouched, then replication is not affected (18,55). Interestingly, altering LBS2 to LBS1 has a slight enhancement in the repression, but not in replication (55). It is tempting to postulate that LBS2 might be associated with repression while LBS3 with replication. Nevertheless, further studies are required to dissect such functions *in vivo*. The tandem high- and low-affinity sites are likely to allow LANA to form a flexible tetramer, resulting in an overall stronger binding to LBS1–2 by positive cooperativity. Furthermore, additional positive cooperativity for LANA is achieved by including LBS3 to LBS1–2 and the binding mode changes from single to biphasic, possibly because LANA may need to vary its binding mode with respect to tethering and replication function. The observed rotational flexibility within the kLANA tetramer suggests that it is likely that the longer spacer region between LBS1 and LBS3 would allow more rotational freedom around two bound LANA dimers compared to LBS1 and LBS2.

**Figure 7.**
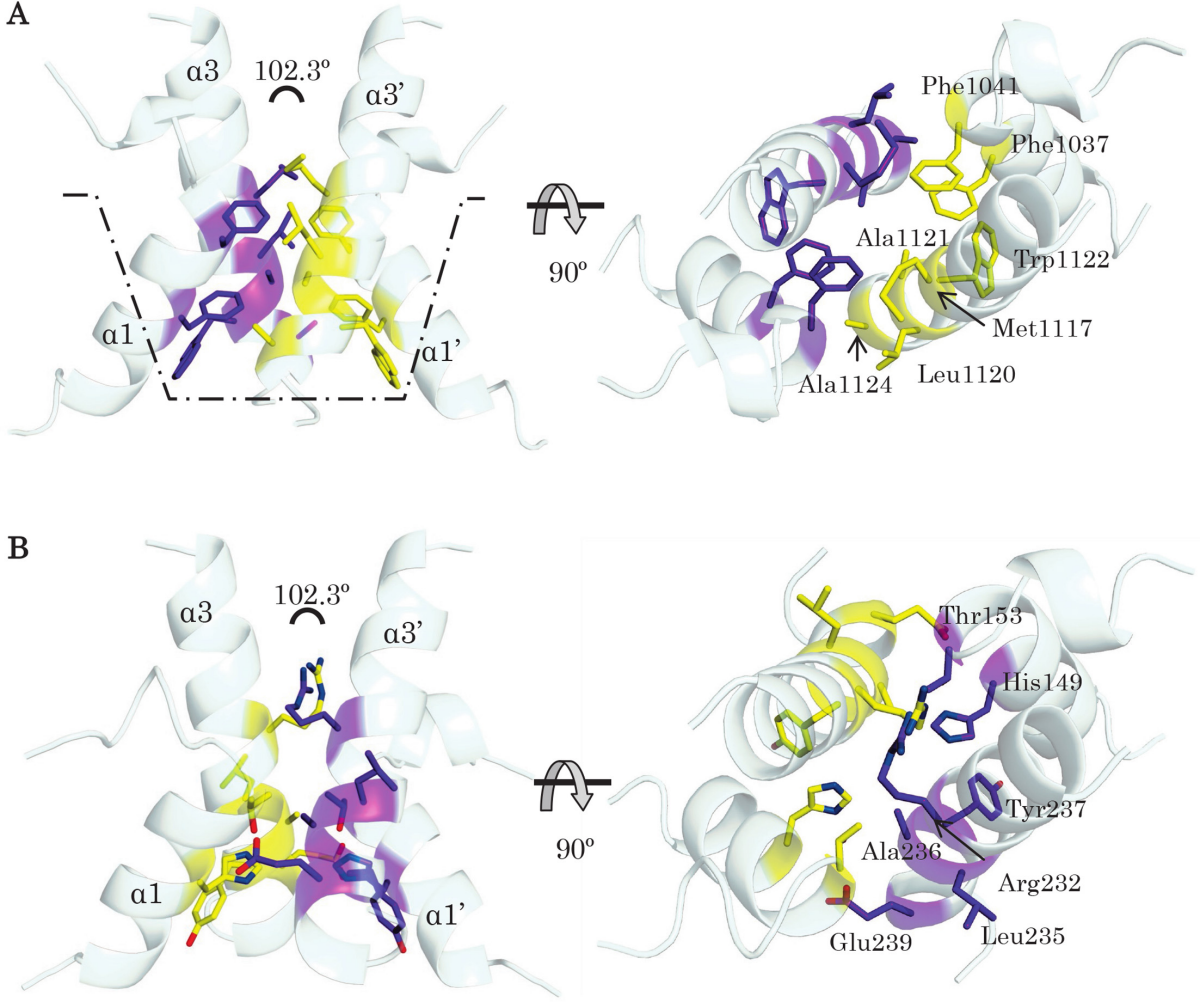
The bend properties of the kLANA tetramer interface. (**A**) The tetramer interface of kLANA DBD with hydrophobic residues (dimer 1, purple sticks and dimer 2 as yellow sticks) are concentrated at one end of the helices creating a hydrophobic bucket (dotted lines) which intrinsically bends the tetramer. (**B**) Superposition of mLANA monomers on the kLANA bent conformation shows that mLANA is able to sterically accommodate a bent conformation, however, the equivalent residues are hydrophilic in nature and lack the driving force to bend the tetramer. Two orthogonal views of the tetrameric interface of LANA are displayed to illustrate the difference in each interface.

The question remains, why does kLANA DBD but not mLANA DBD bend at the dimer–dimer interface? The dimer–dimer assembly interface in kLANA and mLANA DBDs are mediated by the helices α1 and α3 facing the equivalent helices in the second dimer. Overall both kLANA and mLANA DBD bury a similar surface area upon tetramer formation, on average of between 504 Å^2^ and 538 Å^2^ per monomer, respectively. But the gained solvation free energy (Δ^i^*G*) upon kLANA DBD tetramer formation is much larger, Δ*^i^G* of -9.9 kcal/M compared to mLANA Δ*^i^G* of −2.5 kcal/M; demonstrating the dimer–dimer interface is more hydrophobic in kLANA. The core of hydrophobic residues in kLANA is located at one end of both helix α1 (Phe 1037, 1041) and α3 (Met 1117, Leu 1120, Ala 1121, Trp 1122 and Ala 1124), which drive the dimers to intrinsically adopt a bent conformation (Figure 7A). Although mLANA DBD dimers can be superimposed on to the kLANA DBD bent tetramer without major steric clashes (Figure 7B), six out of eight hydrophobic kLANA DBD residues are substituted by polar or charged residues in mLANA DBD, and therefore lack the driving force to adopt a similar conformation (Figures 2B and 7 and Supplementary Figure S4).

Another interesting feature within the kLANA DBD bent tetramer structure is the pivot flexibility at the assembly interface, leading it to adopt three different bend angles observed here in our reported structure and two previous crystal structures (27,28). Comparison of the assembly interface between our structure and the ring structure also demonstrates a rotation around the pivot region, suggesting that these motions are likely to contribute further flexibility for conformational changes that are needed during the TR DNA tethering process. It is likely that, as observed in λcl repressor-asymmetric DNA binding, a rotating or twisting motion at the assembly region could be needed to bring the second LANA molecule to the correct face of the LBS2 DNA site (66).

kLANA DBD achieve this greater flexibility by having smaller hydrophobic alanine residues 1121 and 1124 facing the equivalent residues of the second dimer at the pinnacle of the helices α3 (Figures 6B and 7A). Additionally, mutating alanine 1121 to glutamine reduced the binding affinity to kLBS1–2 DNA (Figure 5A) and consequently episome replication and speckle formation (27). On the contrary, mutating a large charged residue to a small hydrophobic residue from the N-termini of helix α3, lysine 1113 and 1114 to alanine, promotes better binding to LBS DNA and showed differences in the bending of DNA compared to wild-type of about 25° (67), possibly due to increased available space and hydrophobicity at the dimer–dimer interface. Similar changes within hydrophobic residues from phenylalanine to alanine of residues 1037 and 1041 in helix α1 reduced DNA binding and impaired replication and latency (28). These observations highlight the importance of this dimer–dimer interface in KSHV LANA and even the slightest change at this interface has an effect on the conformation of tetramer and consequently octamer assembly with drastic results in the ability of kLANA to promote latent infection. Unlike the kLANA assembly interface, mLANA is more rigid and the residues at the interface are hydrophilic and bulky to allow any flexibility. This LANA interface could be targeted for designing new drugs that aim to disrupt this assembly mechanism.

The closely related EBV EBNA1 protein is also proposed to bend and distort the DNA at the origin of replication (oriP) (68). In the crystals of EBNA1, both the free and DNA bound states did not reveal any tetramer assembly-interface. Yet Bochkarev *et al*., postulated that the EBNA1 protein is likely to form a bent tetramer structure mediated by the flanking helices (69). In order for EBNA1 to adopt this assembly interface, the α3 helices of each dimer have to be in close proximity, but this is hampered by acidic residues at the face of α3 helix (Asp 577, 581 and Glu 573) (see Supplementary Figure S5A). However, like mLANA, EBNA1 can be modelled to adopt a linear tetramer structure, by mainly using residues from α1 helix (see Supplementary Figure S5B). Within the EBV origin of replication, two pairs of additional EBNA1 binding sites are separated by 32 bp (70). Therefore, it is possible that binding between tetramers may induce a bend in DNA. This suggests that mLANA, which binds DNA linearly, may need a second region of binding, similar to EBNA1, if it is to bend DNA. Previous mLANA DNA-footprinting experiments have suggested potential additional binding sites (21).

Analysis based on a structure-based sequence alignment (Supplementary Figure S4) of Herpesvirus DBDs highlight only RFHVMn LANA (A1XYV9) as a likely candidate to form a hydrophobic pivot causing bend at dimer–dimer interface as observed in kLANA. Both MHV-68 LANA and EBV EBNA1 lack these hydrophobic residues. It is unclear whether or not other herpesvirus LANA proteins show divergent variation and adopt either bent or linear tetramer form.

What is the *in vivo* relevance of bent versus linear conformation? The redistribution of LANA to dots in the presence of episomes (Supplementary Figure S6) is most likely due to high affinity, cooperative binding to multiple LBSs within each episome. Each kTR element contains three adjacent LBS's (15,18) and each mTR element may also contain up to three LBSs (21). Although the plasmids initially transfected into LANA expressing cells contained eight kTR or four mTR elements, there is strong selection for recombinant episomes containing higher numbers of TR elements as cells are expanded in culture (14,20,71–72). The KSHV genome contains ∼40 TR elements and it is likely that the recombinant episomes contain a similar number of TRs. The precise nature and arrangement of LANA molecules within the concentrated dots in cell nuclei is unknown, but may be related to oligomerization of LANA dimers and tetramers. It is notable that despite mLANA's linear DNA binding and kLANA's bent binding to DNA, both form similar concentrated dots with episomes.

The consequence of dot formation is that it could lead to a multi-functional platform to enable LANA to carry out its pleiotropic effects and increase its avidity for otherwise weak host protein interactions. The ability of kLANA to adopt a bent conformation suggests a unique strategy for KSHV function(s) within its human host. The bent conformation is consistent with initiation of a pre-initiation complex at the origin of replication by recruiting ORCs to TR DNA (23,73) followed by recruiting Toposiomerase IIβ and loading PCNA to enhance replication (24,74). The linear assembly of mLANA suggests a different approach is used by herpesvirus MHV-68.

In conclusion, we have identified and found the structural basis for how kLANA and not mLANA is able to bend the LBS1–2 DNA. The versatility of LANA's functions could be attributed to the different observed quaternary structures that are linked to a specific phase of viral latency, tethering or replication in herpesviruses. It is surprising that the closely related KSHV and MHV-68 viruses evolved differently to bind TR viral DNA. Further work is needed to dissect and shed more light whether each of the observed assemblies has any relevance to a specific function *in vivo*.

## ACCESSION NUMBERS

The atomic coordinates and structure factors of kLANA DBD have been deposited in the RCSB Protein Data Bank with accession code 5A76. The solution scattering data and models of LANA and LANA DNA complexes have been deposited in the small-angle scattering biological data bank with accession codes SASDAQ8, SASDAS8, SASDAP8, SASDAM8, SASDAR8 and SASDAN8.

## Supplementary Material

SUPPLEMENTARY DATA
