# Supplementary material for: KSHV but not MHV-68 LANA induces a strong bend upon binding to terminal repeat viral DNA
Source: Nucleic Acids Res. 2015 Sep 30;43(20):10039–54. doi: 10.1093/nar/gkv987 (PMC4787769; doi:10.1093/nar/gkv987)
Supplement: SUPPLEMENTARY DATA [file supp_43_20_10039__index.html]

KSHV but not MHV-68 LANA induces a strong bend upon binding to terminal repeat viral DNA — SUPPLEMENTARY DATA 

# KSHV but not MHV-68 LANA induces a strong bend upon binding to terminal repeat viral DNA

## SUPPLEMENTARY DATA

- SUPPLEMENTARY DATA
