# Supplementary material for: KSHV but not MHV-68 LANA induces a strong bend upon binding to terminal repeat viral DNA
Source: Nucleic Acids Res. 2015 Sep 30;43(20):10039–54. doi: 10.1093/nar/gkv987 (PMC4787769; doi:10.1093/nar/gkv987)
Supplement: SUPPLEMENTARY DATA [file supp_gkv987_Supplementary-revised.pdf]

SUPPLEMENTARY METHODS

Fluorescence microscopy of LANA dots

BJAB cells stably expressing LANA (1) were transfected with pk8TR by electroporation. pk8TR contains eight copies of the KSHV terminal repeat unit (TR) cloned into pRep9 (Invitrogen) (2). MEF cell lines stably expressing C3×Flag tagged mLANA driven by its native promoter with m4TR (four copies of the MHV-68 TR unit) were generated by transfection with mLANAF-m4TR (3), using Effectene transfection reagent (Qiagen). 48h after transfection, cells were re-seeded and selected for G418 resistance (600 µg/ml for kLANA, 1,000 µg/ml for mLANA) (Gemini). G418-resistant clones were picked for expansion and then harvested for immunofluorescence. BJAB or MEF (after trypsin digestion) cells were spread onto 10-well slides and air dried at 37ºC. Cells were fixed for 10 min in 4% paraformaldehyde (Polysciences) in phosphate-buffered saline (PBS) and permeabilized for 5min with 0.5% Triton in PBS. kLANA was detected with an anti-LANA monoclonal antibody (1:500; Advanced Biotechnologies), followed by an Alexa Fluor 488-conjugated anti-rat antibody (1:1,000; Molecular Probes) and Flag-tagged mLANA was detected with the M2 anti-FLAG monoclonal antibody (1:500, Sigma) followed by an anti-mouse–Alexa Fluor 488 (1:500, Molecular Probes). Cells were counterstained with propidium iodide (1µg/ml; Molecular Probes) for DNA detection. Microscopy was performed using a Zeiss Axioskop microscope.

SUPPLEMENTARY TABLE 1: Bend and rotational angles

Crystal structures	Bend angle (º)	Reported bend angles (º)
**Octamer (2YQ0)**	95.8	90 [4]
**Decamer (2YPY)**	74.1	72 [4] and 110 [5]
**Spiral (4UZC)**	102.4	
**Non-ring (5A76)**	102.3	

Bend angles were calculated between two dimers of tetramer with their centre of mass as a reference point using a pymol script (draw_rotation_axis). The bend angle and axis are denoted in Figure S7.

Rotational angle helix-3 (º)	Decamer (2YPY)	Spiral (4UZC)	Non-ring (5A76)
**Octamer (2YQ0)**	22.8	12.9	26.2
**Decamer (2YPY)**		23.7	34.1
**Spiral (4UZC)**			13.6

Rotational angles were calculated by superpositioning dimer1 of all four tetramer structures and measuring the rotation angle between the respective helix-3 of dimer2 using a pymol script (angle_between_helices). The angles between the helices are denoted in Figure S8.

SUPPLEMENTARY FIGURES

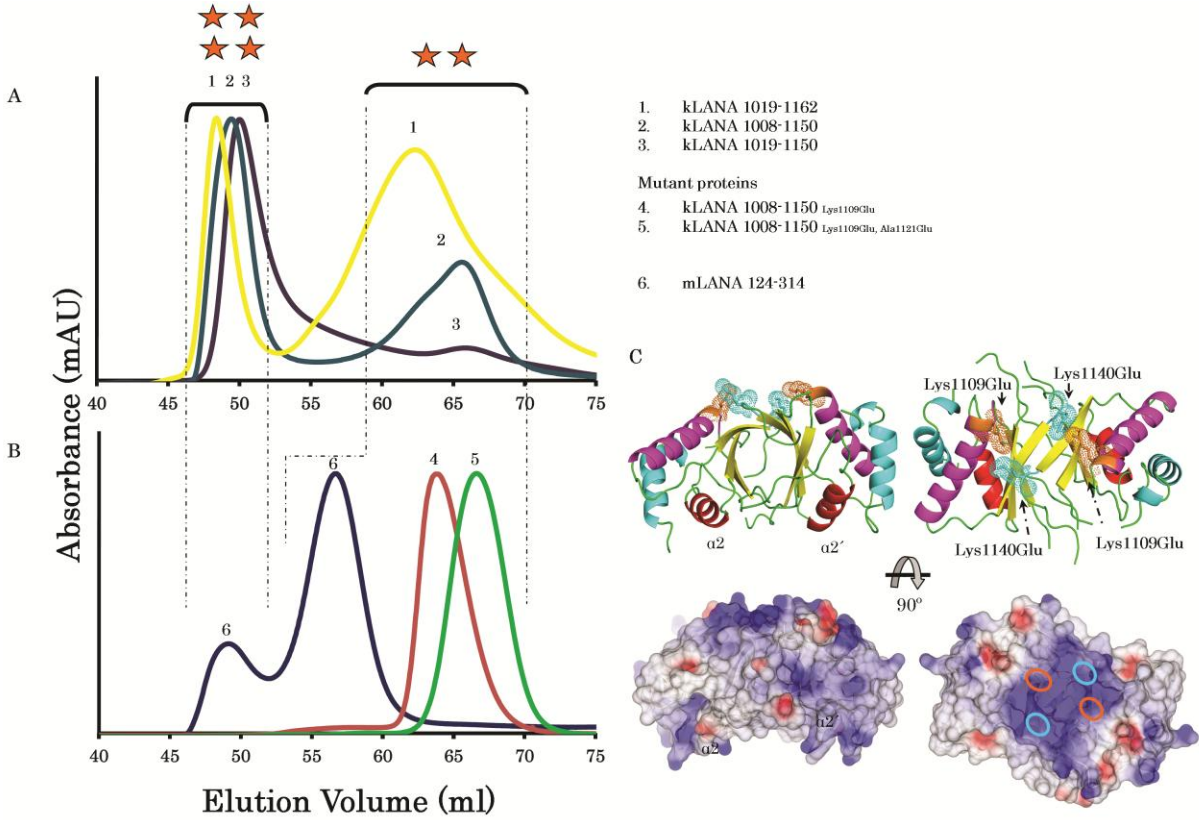

Figure S1. Size exclusion analysis of LANA DBD oligomerization. (A) Wild-type kLANA DBDs truncations showed a predominant tetrameric species with various proportions of dimer species also present independent of high salt (500 mM NaCl). **(B)** kLANA 1008-1150 $_{Lys1109Glu}$ mutant protein showed only a homogenous dimer species. Similarly the pivot mutant kLANA 1008-1150 $_{Lys1109Glu,\ Ala1121Glu}$ behaved as a dimer. Wild-type mLANA 124-314 is predominantly dimer with a small fraction of tetramer also present. Both mutant kLANA and wild-type mLANA were stable in lower salt (250 mM NaCl). (C) kLANA dimer structure in two orientations (upper panel) and with electrostatic potential (lower panel). The helix α2 coloured in red responsible for DNA interaction is at the bottom side, while the mutant residues (Lys1109Glu and Lys 1140Glu) are at the top side of the dimer shown in stick and dotted surface (upper panel). The point mutation introduced a negative charge at the positive patch; Lys1109Glu (orange circle), but not Lys1140Glu (cyan circle), increased stability of the protein.

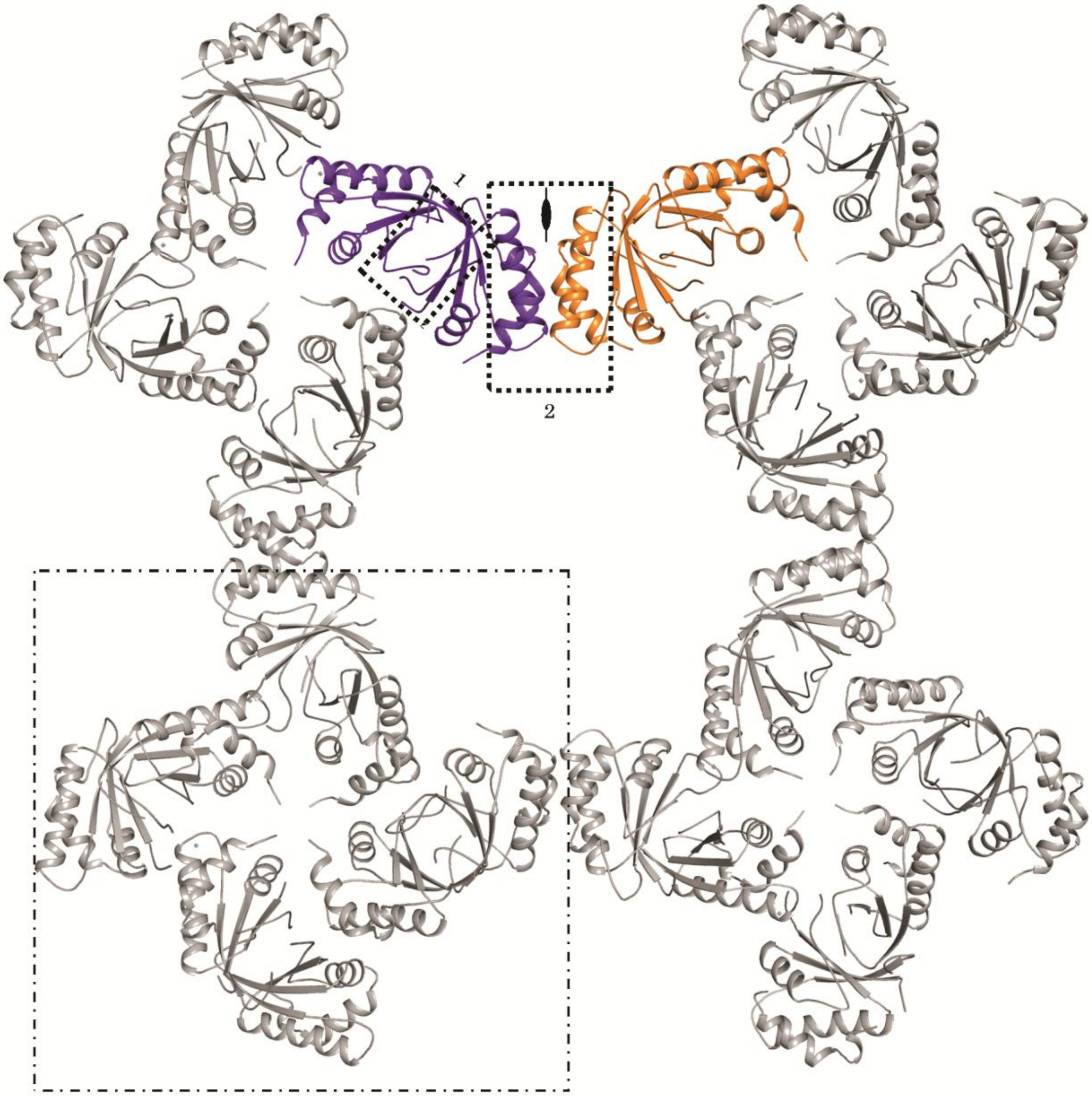

Figure S2. The structure of the kLANA DBD tetramer forms a non-ring conformation. The asymmetric unit contains 4 molecules boxed by dotted lines. The dimer interface is labelled 1. Two dimer molecules coloured in purple and orange are shown to form a bent tetramer at the two fold axis. No ring formation through the assembly interface (dotted box 2) was observed in the crystal packing as shown by the packing of asymmetric units in grey.

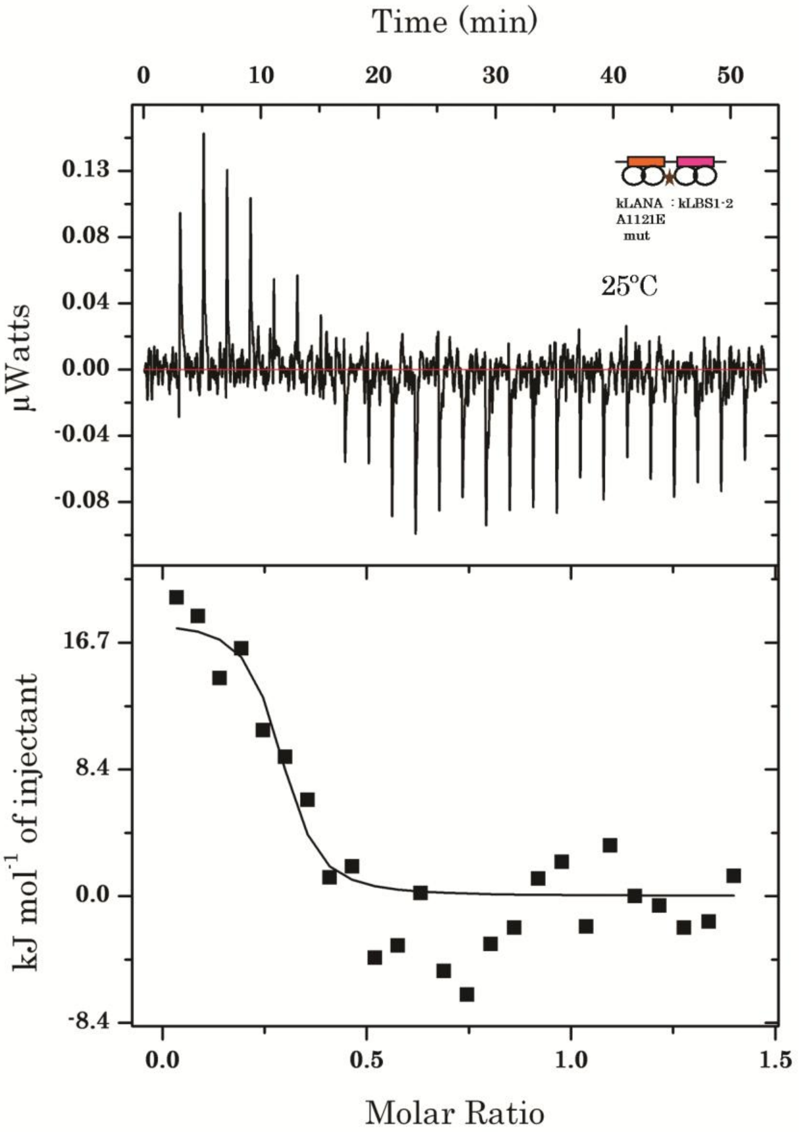

Figure S3. ITC analysis of the pivot mutation on kLBS1-2 DNA binding. Unlike wild type kLANA DBD, the pivot mutant produced less measurable energy at 25 ºC. Although the signal is close to background we were able to fit a one-site binding model. Increasing the protein and DNA concentrations were not successful to enhance the signal

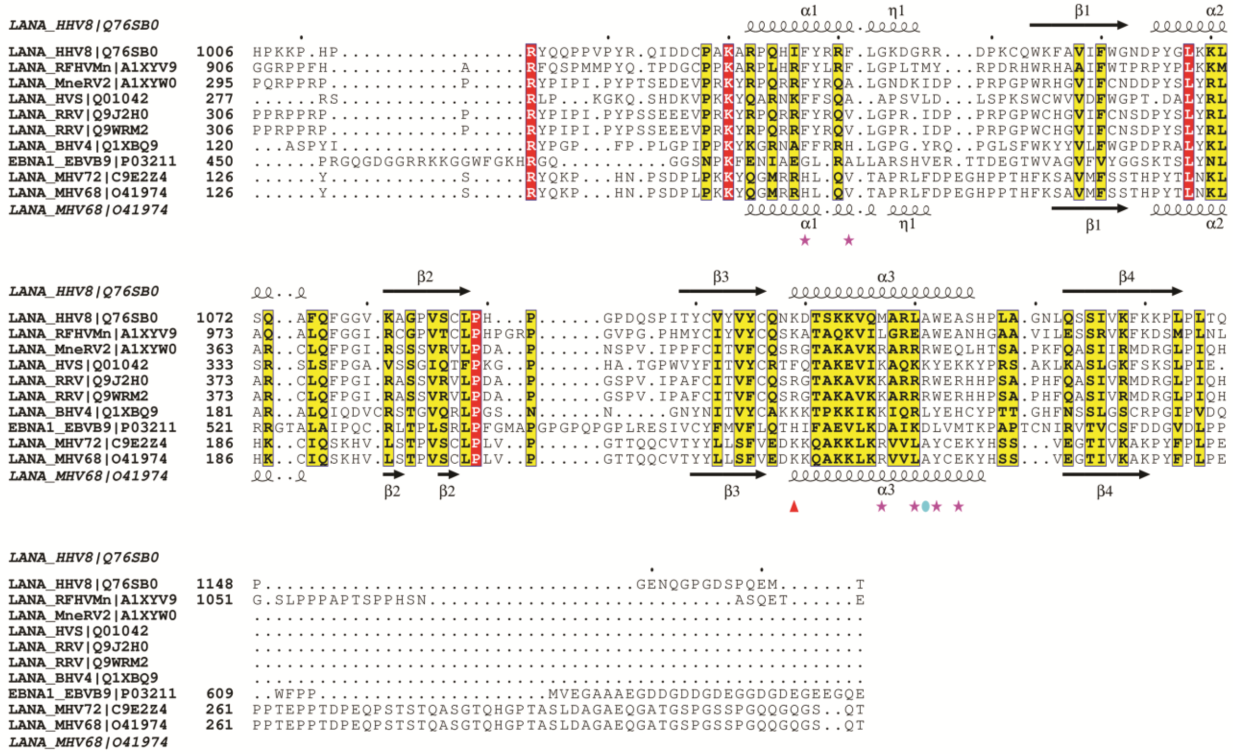

Figure S4. Structure-based sequence alignments of OBP LANA and EBNA1 DNA binding domains were performed using Expresso (6) and displayed using ESPript 3.0 (7). Secondary structure elements of kLANA (from PDBID: 4K2J) and mLANA DBDs (PDBID: 4BLG) are shown above and below the alignments, respectively. Conserved residues are shown in white on a red background and similar residues are highlighted in yellow. The residues of the dimer-dimer assembly interface are marked as pink stars. The Ala 1121 residue at the pivot region is marked by a cyan circle while the Lys1109Ala solubility mutant is marked by a red triangle.

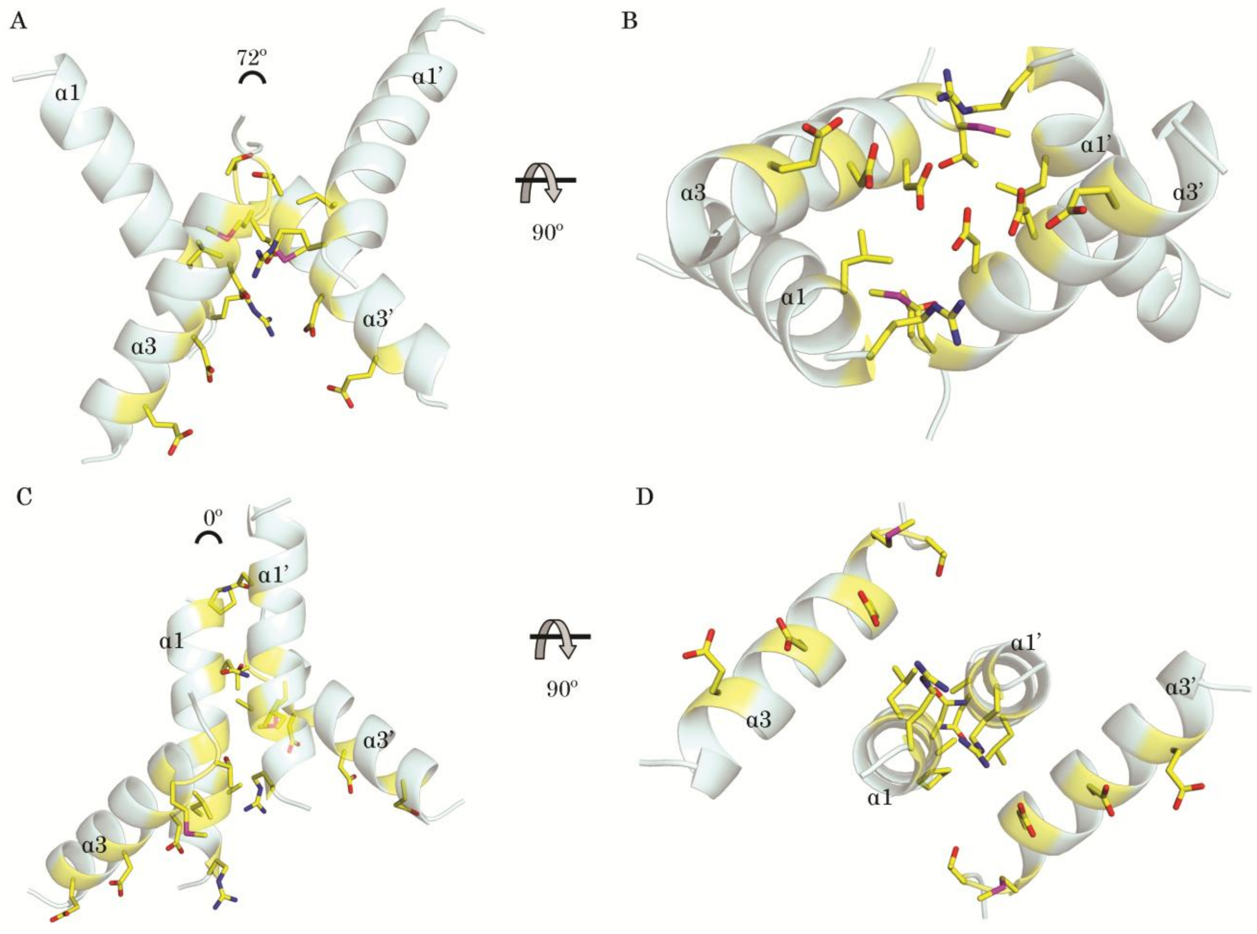

Figure S5. Modelling of EBNA1 adopting bent or linear conformation at the assembly tetramer interface.

(A) Superposition of EBNA1 monomers on the kLANA bent conformation shows that EBNA1 is not able to accommodate a bend conformation, the acidic residues from each monomer of helix α3 would exert a repulsive force. **(B)** Superposition of EBNA1 monomers on the mLANA linear conformation shows some steric clashes with residues from helix α1, but with some rearrangements EBNA1 could accommodate this linear tetramer conformation. At the centre of α1 helix small hydrophobic residues (Gly 484 and Ala 487) would allow any required flexibilities at this interface. Two orthogonal views of the tetrameric interface of LANA are displayed to illustrate the difference in each interface.

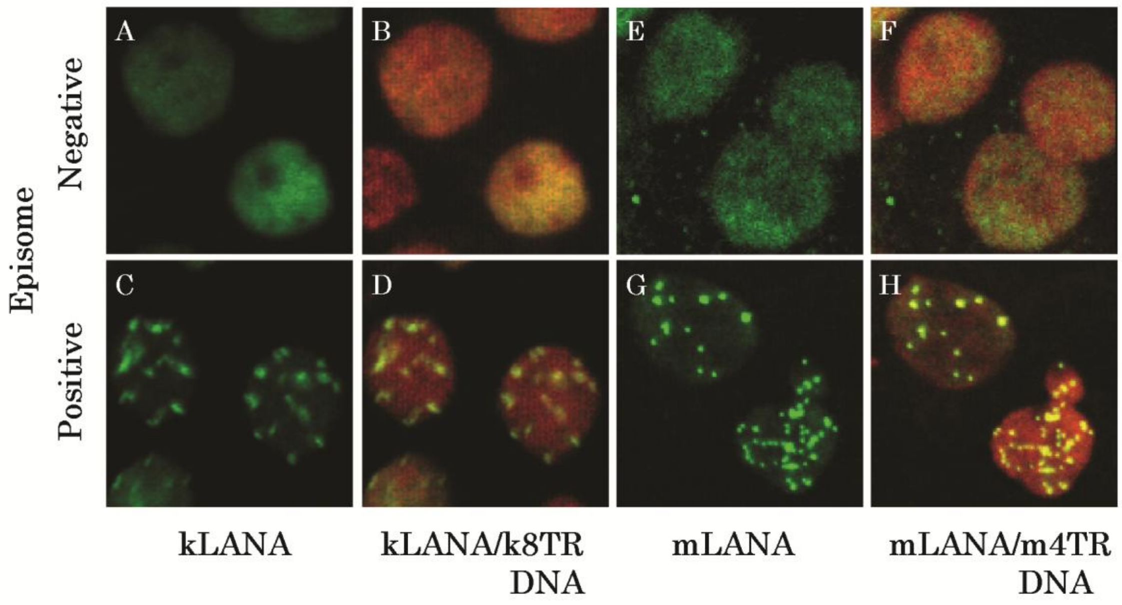

Figure S6. LANA concentrates to dots in the presence of viral episomes.

kLANA (green) was detected in kLANA expressing BJAB cells in the absence **(A and B)** or presence **(C and D)** of episomal DNA. Similarly, mLANA (green) was detected in MEF cells in the absence **(E and F)** or presence **(G and H)** of episomal DNA. DNA (red) was counterstained with propidium iodide. Overlay of green and red generates yellow. Magnification x630.

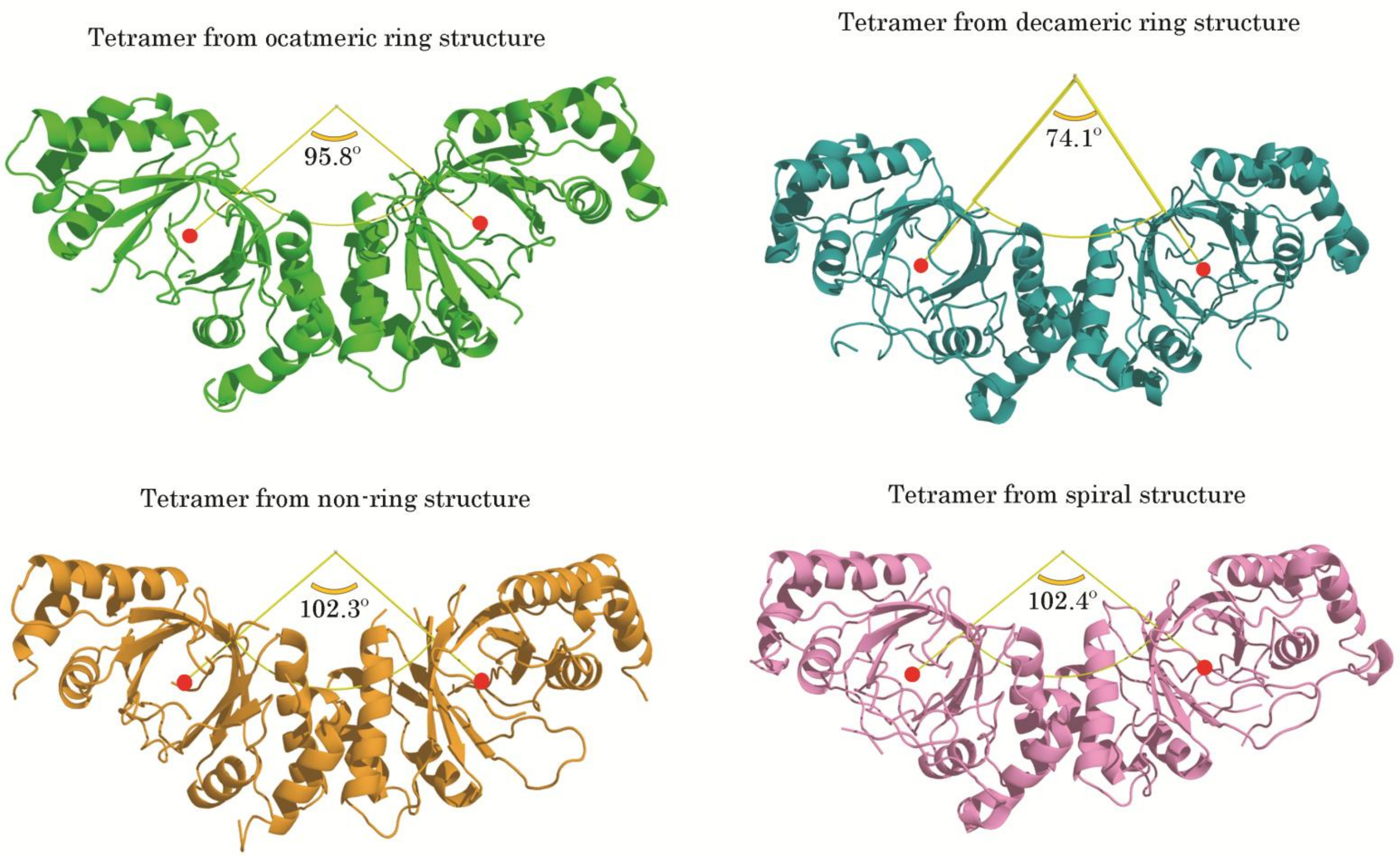

Figure S7. kLANA-bend angles between dimers.

Crystal structures of kLANA-DBD have been solved so far in four different crystalline environments; with each exhibiting a varying bend angles at the assembly interface (dimer-dimer). The bend angles were calculated between two dimers of tetramer with their centre of mass as a reference point (red circle). Tetramers are represented in ribbon from Octamer-ring structure (2YQ0; green), Decamer-ring structure (2YPY; teal), Non-ring structure (5AK6; orange) and Spiral (4UZC; magenta).

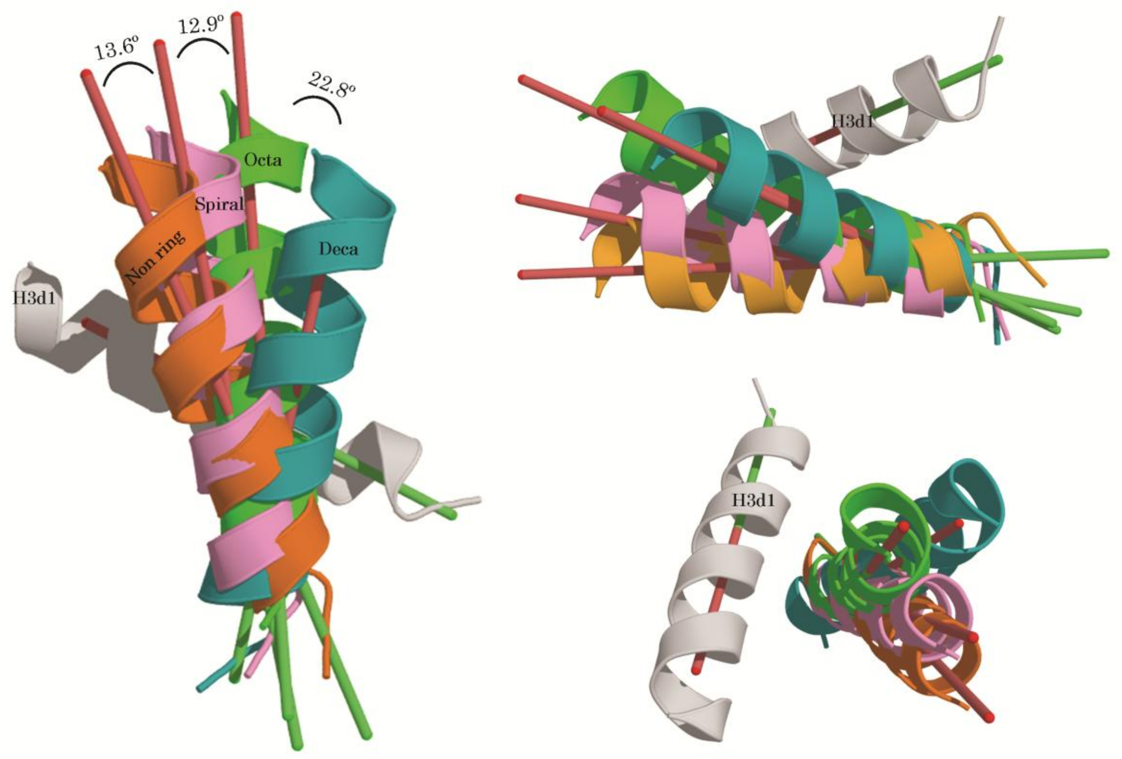

Figure S8. kLANA-rotational angles between dimers.

In addition to the bend angles (Figure S7), kLANA assembly interface includes a rotational angle between dimers of tetramer. Rotational angles were calculated by superpositioning dimer1 of all tetramer and measuring the angle between the helix-3 of respective structures in dimer2. Dimer1 superimpose well (Figure 3B), only one of the Helix-3 from dimer1 (H3d1) is represented for clarity. Helix-3 of dimer2 from Octamer-ring structure (Octa; 2YQ0; green), Decamer-ring structure (Deca; 2YPY; teal), Non-ring structure (5AK6; orange) and Spiral (4UZC; magenta) are represented. In combination with bend (Figure S7) and rotational angle, kLANA is able to achieve various higher oligomers.

SUPPLEMENTARY REFERENCES

1. Vázquez,E., Carey,V. and Kaye,K. (2013) Identification of Kaposi's Sarcoma-Associated Herpesvirus LANA Regions Important for Episome Segregation, Replication, and Persistence. *J. Virol.*, **87**, 12270–12283.

2. Barbera,A.J., Ballestas,M.E. and Kaye,K.M. (2004) The Kaposi's sarcoma-associated herpesvirus latency-associated nuclear antigen 1 N terminus is essential for chromosome association, DNA replication, and episome persistence. *J. Virol.*, **78**, 294–301.

3. Habison,A.C., Beauchemin,C., Simas,J.P., Usherwood,E.J. and Kaye,K.M. (2012) Murine gammaherpesvirus 68 LANA acts on terminal repeat DNA to mediate episome persistence. *J. Virol.*, **86**, 11863–76.

4. Hellert,J., Weidner-Glunde,M., Krausze,J., Richter,U., Adler,H., Fedorov,R., Pietrek,M., Rückert,J., Ritter,C., Schulz,T.F., *et al.* (2013) A structural basis for BRD2/4-mediated host chromatin interaction and oligomer assembly of Kaposi sarcoma-associated herpesvirus and murine gammaherpesvirus LANA proteins. *PLoS Pathog.*, **9**, e1003640.

5. Domsic,J.F., Chen,H.-S., Lu,F., Marmorstein,R. and Lieberman,P.M. (2013) Molecular basis for oligomeric-DNA binding and episome maintenance by KSHV LANA. *PLoS Pathog.*, **9**, e100367.

6. Armougom,F., Moretti,S., Poirot,O., Audic,S., Dumas,P., Schaeli,B., Keduas,V., Notredame,C. and Notredame,C. (2006) Expresso: Automatic incorporation of structural information in multiple sequence alignments using 3D-Coffee. *Nucleic Acids Res.*, **34**, 604–608.

7. Robert,X. and Gouet,P. (2014) Deciphering key features in protein structures with the new ENDscript server. *Nucleic Acids Res.*, **42**, 320–324.
